# A cost-effective, ionically conductive and compressible oxychloride solid-state electrolyte for stable all-solid-state lithium-based batteries

**DOI:** 10.1038/s41467-023-39522-1

**Published:** 2023-06-27

**Authors:** Lv Hu, Jinzhu Wang, Kai Wang, Zhenqi Gu, Zhiwei Xi, Hui Li, Fang Chen, Youxi Wang, Zhenyu Li, Cheng Ma

**Affiliations:** 1 Hefei National Research Center for Physical Sciences at the Microscale, CAS Key Laboratory of Materials for Energy Conversion, Department of Materials Science and Engineering, University of Science and Technology of China, Hefei, 230026 Anhui China; 2 Key Laboratory of Precision and Intelligent Chemistry, University of Science and Technology of China, Hefei, 230026 Anhui China; 3 National Synchrotron Radiation Laboratory, Hefei, 230026 Anhui China

**Keywords:** Batteries, Inorganic chemistry, Materials for energy and catalysis, Electrochemistry, Energy

## Abstract

To enable the development of all-solid-state batteries, an inorganic solid-state electrolyte should demonstrate high ionic conductivity (i.e., > 1 mS cm^−1^ at 25 °C), compressibility (e.g., > 90% density under 250−350 MPa), and cost-effectiveness (e.g., < $50/kg). Here we report the development and preparation of Li_1.75_ZrCl_4.75_O_0.5_ oxychloride solid-state electrolyte that demonstrates an ionic conductivity of 2.42 mS cm^−1^ at 25 °C, a compressibility enabling 94.2% density under 300 MPa and an estimated raw materials cost of $11.60/kg. As proof of concept, the Li_1.75_ZrCl_4.75_O_0.5_ is tested in combination with a LiNi_0.8_Mn_0.1_Co_0.1_O_2_-based positive electrode and a Li_6_PS_5_Cl-coated Li-In negative electrode in lab-scale cell configuration. This all-solid-state cell delivers a discharge capacity retention of 70.34% (final discharge capacity of 70.2 mAh g^−1^) after 2082 cycles at 1 A g^−1^, 25 °C and 1.5 tons of stacking pressure.

## Abstract (web summary)

Ionic conductivity > 1 mS cm^−1^ at 25 °C, compressibility enabling > 90% density at 250 − 350 MPa, and cost < $50/kg are desirable for inorganic solid-state electrolytes. Here, the authors report Li_1.75_ZrCl_4.75_O_0.5_ as a solid-state electrolyte capable of satisfying these requirements simultaneously.

## Introduction

Identifying appropriate solid electrolytes is the first step toward the construction of safe, energy-dense all-solid-state Li batteries (ASSLBs)^1–4^. Ideally, the solid electrolyte should excel simultaneously at ionic conductivity^2,5,6^, compressibility^7,8^, electrochemical stability^8–11^, humidity tolerance^12–14^, and cost-effectiveness^12,15^. Fortunately, some of these characteristics are not absolutely necessary. For example, if appropriate coating can be applied to the electrodes, solid electrolytes with relatively low intrinsic electrochemical stability are acceptable^16–22^. If the workflow of mass production can be designed to avoid moisture exposure of solid electrolytes, they do not have to show high humidity tolerance^3,16^. With such characteristics excluded, the solid electrolyte still needs to exhibit reasonable performance in three aspects: ionic conductivity, compressibility, and cost-effectiveness.

These characteristics play different roles in ASSLBs. The ionic conductivity is important not only because it determines the ion transport efficiency^2^, but also because it indirectly influences the energy density^5^. If the solid electrolyte is highly ionically conductive, it only needs to occupy a small fraction of the composite electrode to realize efficient ion transport, and thus higher loading of active materials may be used^5,23^. To this end, an ionic conductivity at 25 °C at the level of 0.1 mS cm^−1^ would not suffice; instead, ionic conductivities above 1 mS cm^−1^ are desired^5,24^. As for the compressibility, it determines the quality of positive electrode-electrolyte contact^9,25,26^; higher compressibility allows the solid electrolyte to cover larger area of the active material particles under pressure. Recently, it is suggested that the solid electrolyte should preferably be compressible enough to make the cold-pressed pellet fabricated under 250−350 MPa more than 90% dense^25^. Last but not least, the material cost of solid electrolytes must not exceed $50/kg^27,28^. Otherwise ASSLBs would not be competitive against the present commercial Li-ion batteries in the market.

Unfortunately, none of the reported inorganic solid electrolytes can simultaneously meet these three requirements (ionic conductivity above 1 mS cm^−1^ at 25 °C, sufficiently high compressibility to realize over 90% density under 250−350 MPa, and material cost below $50/kg). The present inorganic solid electrolytes may be divided into three categories^2,8^: oxides, sulfides, and halides. As brittle solids, the oxides cannot meet the requirement on compressibility^4,29^. In comparison, the sulfides and halides are both compressible under pressure, and are also relatively easy to reach high ionic conductivities^2,8^. Nevertheless, most of them are not cost-effective^12^. The cost of sulfide solid electrolytes arises mainly from the raw material Li_2_S^12^. According to a recent report^12^, the unit price of Li_2_S in 1000 kg purchase is as high as $654.18/kg, and its weight fraction in the raw materials of most sulfide solid electrolytes are above 30%. Therefore, even if other chemicals used for synthesis are free, the cost would still exceed $196.25/kg, much higher than the $50/kg threshold. As for halides, most of them need to be synthesized using the rare earth chlorides and/or indium chloride, whose prices range between $320.33/kg (YCl_3_) and $28635.19/kg (LuCl_3_)^12^, making the corresponding solid electrolytes cost no lower than $196.31/kg (Li_3_YCl_6_). The only exception is the recently reported Li_2_ZrCl_6_, but its ionic conductivity is below 1 mS cm^−1^ at 25 °C^12,15^. Beyond the cost, there is also concern about compressibility; although the sulfide and halide solid electrolytes are not rigid like oxides, few of them are compressible enough to reach densities above 90% under 250−350 MPa; Li_6_PS_5_Cl is only 75.4% dense under 250 MPa^30^, and Li_3_YCl_6_ is 76−79% dense under 350 MPa^31^. More in general, none of the state-of-the-art oxide, sulfide, and halide solid electrolytes can satisfy the three aforementioned requirements simultaneously.

Here, we report an oxychloride solid electrolyte (i.e., Li_1.75_ZrCl_4.75_O_0.5_) that meets all these three requirements. It shows an ionic conductivity of 2.42 mS cm^−1^ at 25 °C, a density of 94.2% under 300 MPa, and a material cost of $11.60/kg. The fulfilling of these practical requirements translated in stable long-term Li-based battery operation. Indeed, when the Li_1.75_ZrCl_4.75_O_0.5_ is tested in combination with a LiNi_0.8_Mn_0.1_Co_0.1_O_2_-based positive electrode and a Li_6_PS_5_Cl-coated Li-In negative electrode, the lab-scale cell demonstrates a discharge capacity retention of 70.34% (final discharge capacity of 70.2 mAh g^−1^) after 2082 cycles at 1 A g^−1^, 25 °C and 1.5 tons of stacking pressure.

## Results and discussion

### Synthesis, crystal structure, and ionic conductivity of Li_1.75_ZrCl_4.75_O_0.5_

**Fig. 1:**
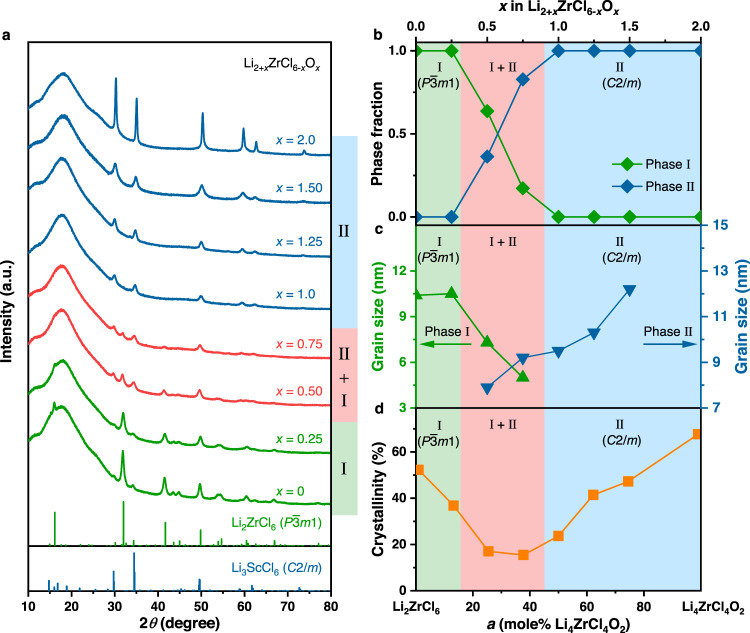
Composition-dependent phase evolution in Li_2+*x*_ZrCl_6-*x*_O_*x*_. **a** XRD patterns of the mechanochemically synthesized Li_2+*x*_ZrCl_6-*x*_O_*x*_. The broad hump below 30° comes from the Kapton film used to prevent air exposure during measurement. No smoothening was conducted to any of the diffraction data displayed here. **b**−**d** Variation of phase fractions (**b**), grain sizes (**c**), and crystallinity (**d**) with composition. The data were acquired from Rietveld refinement.

In literature, the search of high-performance solid electrolytes has been conducted primarily among single-phase materials and their glass ceramics^2,4,8^, while those containing more than one distinct crystalline phase were barely explored. Nevertheless, the latter class of materials is more versatile than the former in many different research fields, such as piezoelectricity^32^, electrocaloric effect^33^, and magnetoristriction^34^, because the coexistence of multiple energetically comparable states often makes the material more responsive to external stimuli^32^. According to this principle, the “multi-phase” status might possibly enhance the properties relevant to solid electrolytes as well. Based on this assumption, the present study adopts an unconventional strategy to design the solid electrolytes: the focus is placed on the materials where multiple crystalline phases coexist, rather than the single-phase materials or their glass ceramics. To begin with, aliovalent O^2−^ doping was conducted to induce structural phase transitions in Li_2_ZrCl_6_^12,15^. Figure 1a displays the X-ray diffraction (XRD) patterns for a series of mechanochemically synthesized Li_2+*x*_ZrCl_6-*x*_O_*x*_ materials. The compositions with relatively low O content (*x* ≤ 0.25) are phase-pure and isostructural to the unmodified Li_2_ZrCl_6_ with the $P\bar{3}m1$ symmetry^12^. This $P\bar{3}m1$ phase will be referred to as Phase I below. Further increase of the O content leads to the emergence of another crystalline phase (referred to as Phase II below). It coexists with Phase I between *x* = 0.5 and 0.75, and eventually Phase II becomes the only identifiable phase at *x* ≥ 1.0. The characteristic Bragg reflections of Phase II suggest that it is isostructural to Li_3_ScCl_6_ with the *C2/m* symmetry^35^. When the Rietveld refinement was conducted using the $P\bar{3}m1$ and *C2/m* structural models above for Phase I and Phase II, respectively, all the compositions show excellent agreement between the observed and calculated curves (Supplementary Figs. 1−8; refined structures summarized in Supplementary Tables 1−8). Besides, as expected, the refinement results also indicate that Phase II is continuously consuming Phase I with increasing *x* in the two-phase region (Fig. 1b). According to these results, our goal of inducing phase transitions in Li_2_ZrCl_6_ has been successfully achieved by the aliovalent O^2−^ doping. With the occurrence of such composition-induced phase evolution confirmed, an alternative formula, (1−*a*)Li_2_ZrCl_6_−*a*Li_4_ZrCl_4_O_2_ (*a* = *x*/2, 0 ≤ *x* ≤ 2), will also be used in Fig. 1 and the discussion below; although not as brief as Li_2+*x*_ZrCl_6-*x*_O_*x*_, it allows the compositions to be comprehended from the perspective of a binary phase diagram.

As mentioned above, the reason for exploring compositions with the coexistence of multiple crystalline phases lies in the possibility that they might be more responsive to the external stimuli. In fact, this behavior is already reflected in the XRD patterns. As shown in Fig. 1a, the Bragg reflections of the two-phase compositions, i.e., the ones with *x* = 0.50−0.75 (*a* = 25−37.5%), are much more diffuse and weaker than the single-phase ones; the further away the compositions are from this two-phase region, the sharper and stronger the diffraction peaks would become. The diffuse and weak diffraction signals usually entail that the crystallites are small^36,37^. Based on the peak widths, the grain sizes can be estimated using Rietveld refinement, and the results are compared in Fig. 1c. Among these data, the one for the composition with *x* = 2.0 (*a* = 100%) is not displayed, because the sharpness of its diffraction peaks (Fig. 1a) suggests that the grain size is larger than the detectable limit of XRD (below 60 nm^37^). With this composition excluded, the results in Fig. 1c disclose a clear trend: the grain sizes of both Phase I and Phase II minimize within the two-phase region, down to a few nanometers. For mechanochemically synthesized materials, such small crystallite sizes are frequently accompanied by a higher degree of amorphization^36,38^. Although this cannot be reflected directly in the XRD patterns in Fig. 1a due to the broad hump from the Kapton film used to avoid air and moisture contamination during measurements, the crystallinity, i.e., the mass fraction of the crystalline species in the material, may still be estimated by a method proposed by Yasukawa et al.^39^, which is based on Rietveld refinement of XRD patterns for the samples mixed with Ag powder as the internal standard. The results disclose a tendency similar to the variation of grain size: the two-phase compositions exhibit significantly lower crystallinities than the single-phase ones (Fig. 1d). In sharp contrast to the latter (crystallinity up to 67.65%), the former never shows a crystallinity above 20%. That is, for these two-phase compositions, over 80% of their weight is amorphous. The crystallinity decrease associated with the increase of Li_2_O molar ratio during the solid-state electrolyte synthesis can be comprehended by calculating and comparing the global instability index (GII), a parameter that indicates the stability of a crystal structure^40^. Ideally, the sum of bond valences connected to each atom in a given crystal structure should be equal to the absolute value of its oxidation state, but, practically, there would always be some difference. A larger difference entails a lower structural stability; if the root-mean-square difference averaged over all the atoms in a crystal structure, i.e., the GII of this structure, is above 0.20, its stability is questionable^40,41^. Supplementary Fig. 9 shows the GIIs of the crystalline phases in Li_2+*x*_ZrCl_6−*x*_O_*x*_ with different *x*. The displayed values were calculated from the experimentally determined structures (Supplementary Tables 1−4), which may be considered as the most stable forms of the corresponding materials in the crystalline state. Regardless, except for Li_2_ZrCl_6_, such most stable crystalline forms for all the Li_2+*x*_ZrCl_6−*x*_O_*x*_ compositions in Supplementary Fig. 9 exhibit GIIs exceeding 0.20, suggesting that the increase of *x* makes the material more difficult to confine in the crystalline phase. This explains why the use of Li_2_O during the solid-state electrolyte synthesis decreases the crystallinity. On the other hand, when the *x* in Li_2+*x*_ZrCl_6−*x*_O_*x*_ is sufficiently large, the material would begin to resemble Li_4_ZrCl_4_O_2_, the other end-member component that is difficult to become amorphous, like Li_2_ZrCl_6_; from this point on, further increase of *x* would increase the crystallinity instead. With the presence of these two competing tendencies, the lattice of the two-phase compositions in Fig. 1 appears more vulnerable to the intense planetary mill than the single-phase ones; although all the compositions were synthesized using the same milling condition, a considerably higher degree of amorphization occurred in the two-phase compositions, and their grain sizes (below 10 nm) were reduced with respect to those of the single-phase ones too (up to values beyond the detection limit of XRD, i.e., 60 nm^37^). It should be noted that the end-member component Li_2_ZrCl_6_ here relies mainly on the “non-periodic features” such as amorphous species and surface defects to realize facile ion transport^12,15^. Therefore, for the two-phase compositions with lower crystallinity and smaller grains (Fig. 1c−d), higher ionic conductivities might be expected.

**Fig. 2:**
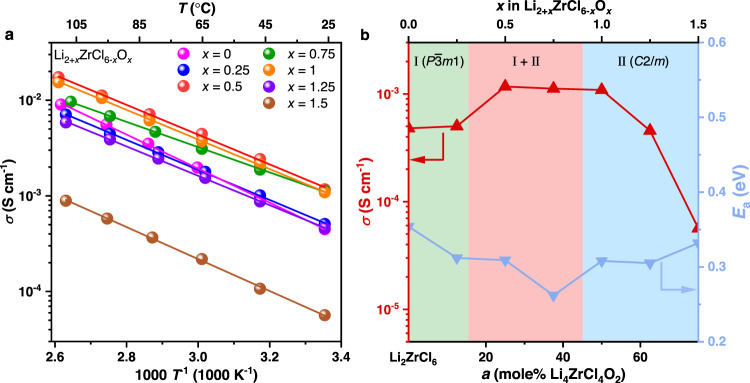
Li-ion transport behavior of Li_2+*x*_ZrCl_6-*x*_O_*x*_. **a** Arrhenius plots of Li_2+*x*_ZrCl_6-*x*_O_*x*_ with different compositions. **b** Variation of the ionic conductivity (*σ*) at 25 °C and the activation energy (*E*_a_) with *x* in Li_2+*x*_ZrCl_6-*x*_O_*x*_.

In order to verify the speculation raised above, the electrochemical impedance spectroscopy (EIS) measurements were conducted. The ionic conductivities of the *x* = 2.0 (*a* = 100%) composition at 25 °C is too low to be measured properly (the resistance of the cold-pressed pellet for measurement exceeds the range of the impedance analyzer), and thus will not be discussed here. As for the other compositions, their ionic conductivities are all above 10^−5^ S cm^−1^ at 25 °C (Supplementary Fig. 10), while the electronic conductivities at the same temperature lie between 10^−10^−10^−9^ S cm^−1^ (Supplementary Fig. 11). Therefore, all these materials may be considered as proper Li-ion conductors. The Arrhenius plots for different compositions are displayed in Fig. 2a, while the corresponding ionic conductivities at 25 °C and the activation energies are compared in Fig. 2b, with the phase regions determined from the XRD patterns in Fig. 1 indicated. As expected, the variation of ion transport behavior follows a similar trend as the grain sizes and crystallinity: the ionic conductivities of the poorly crystallized two-phase compositions are higher than all of the single-phase ones. The most ionically conductive composition is the one with *x* = 0.5 (*a* = 25%), i.e., Li_2.5_ZrCl_5.5_O_0.5_; its ionic conductivity at 25 °C reaches 1.17 mS cm^−1^.

It needs to be emphasized that, similar to the Li_2_ZrCl_6_ material reported earlier^12,15^, the direct contributor to the high ionic conductivity here is the amorphous species (more than 80 wt% in the optimal compositions), rather than the two coexisting crystalline phases (< 20 wt% in the optimal compositions). Selecting a composition in the two-phase region of the phase diagram (Fig. 1d) for synthesis would make it easier to create larger amounts of such Li-ion conductive amorphous species, but the two-phase coexistence itself can by no means facilitate the ion transport. In order to demonstrate this point, we milled the separately synthesized Li_2_ZrCl_6_ and Li_4_ZrCl_4_O_2_ together according to the overall composition of Li_2.5_ZrCl_5.5_O_0.5_, i.e., the most ionically conductive one in the binary Li_2_ZrCl_6_-Li_4_ZrCl_4_O_2_ system (Fig. 2b), in two different ways; one is a physical mixing realized through manual grinding for 30 min, and the other is the intense planetary mill that could increase the amount of the amorphous species (the milling parameters are identical with those used for the mechanochemical synthesis of the individual materials). Since the manual grinding cannot further amorphize the material, the physical mixture may only exhibit a crystallinity between those of the two components, i.e., Li_2_ZrCl_6_ and Li_4_ZrCl_4_O_2_ (crystallinities 52.25% and 67.65%, respectively). Such a high crystallinity (52.25−67.65%), as confirmed by the sharp X-ray diffraction peaks (Supplementary Fig. 12a−c), surpasses that of the directly synthesized Li_2.5_ZrCl_5.5_O_0.5_ (below 20%). In good agreement with the scenario described above, the ionic conductivity of the physical mixture was only 0.214 mS cm^−1^ at 25 °C (Supplementary Fig. 12d), nearly one order of magnitude lower than that of the directly synthesized Li_2.5_ZrCl_5.5_O_0.5_ (1.17 mS cm^−1^ at 25 °C). On the other hand, since the overall composition of the mixture is Li_2.5_ZrCl_5.5_O_0.5_, a two-phase one that is supposed to make the material easier to amorphize, the intense planetary mill should still be able to lower its crystallinity to a level similar to that of the directly synthesized Li_2.5_ZrCl_5.5_O_0.5_, and thus realize a comparable ionic conductivity. Consistent with this inference, the planetary milled mixture does exhibit broad, weak X-ray diffraction peaks (Supplementary Fig. 12a−c), like those of the directly synthesized Li_2.5_ZrCl_5.5_O_0.5_ (Fig. 1a). Correspondingly, the ionic conductivity at 25 °C exceeds 1 mS cm^−1^ again (Supplementary Fig. 12e). These data suggest that simply making the $P\bar{3}m1$ and $C2/m$ phases coexist in the material is not sufficient to induce high ionic conductivities; instead, the efficient ion transport must be realized by creating large amounts of the amorphous species, and this goal is particularly easy to achieve for the compositions lying in the two-phase region of the phase diagram (Fig. 1d).

Although the ionic conductivity improvement here should be attributed mostly to the increase of the Li-ion conductive amorphous species, which exceeds 80 wt% in the optimal compositions presented above (Fig. 1d), the crystalline phases in these materials also exhibit more efficient ion transport than that in Li_2_ZrCl_6_. To demonstrate this point, we compared the Li^+^ migration behavior in the crystalline phases of Li_2_ZrCl_6_ and Li_2.5_ZrCl_5.5_O_0.5_ using the bond valence site energy (BVSE) method developed by Adams et al.^41,42^; the analysis was conducted on the structures obtained from the Rietveld refinement (Supplementary Tables 1 and 3). Consistent with the previous report^12^, the only crystalline phase in Li_2_ZrCl_6_, i.e., the $P\bar{3}m1$ phase, shows three-dimensional ion transport pathways formed by the interconnecting [Li1–Li2–Li1], [Li1–i1–Li1-i2-Li1], and [Li2–i3–Li2] chains (Supplementary Fig. 13a−b), and the effective migration barrier is 0.806 eV (Supplementary Fig. 13c). Unlike Li_2_ZrCl_6_, Li_2.5_ZrCl_5.5_O_0.5_ contains two crystalline phases. One of them exhibits the same structural framework and ion transport pathways as the aforementioned $P\bar{3}m1$ phase in Li_2_ZrCl_6_ (Supplementary Fig. 13d−e), but the differences in the composition and atomic configuration lead to a lower effective migration barrier of 0.748 eV (Supplementary Fig. 13f). As for the other crystalline phase, it is isostructural with the Li_3_ScCl_6_ material showing the *C2/m* symmetry^35^. The Li-ion migration pathways in this structure consist of the [Li1–i1–Li2], [i1–Li1–i1], and [Li1–i1–Li3] chains (Supplementary Fig. 13g−h), and the diffusion is rather facile in all of them. Consequently, the effective migration barrier of the entire three-dimensional network is only 0.411 eV (Supplementary Fig. 13i), much lower than those discussed above. According to these BVSE results, both crystalline phases in Li_2.5_ZrCl_5.5_O_0.5_ can enable more efficient Li-ion migration than that in Li_2_ZrCl_6_.

**Fig. 3:**
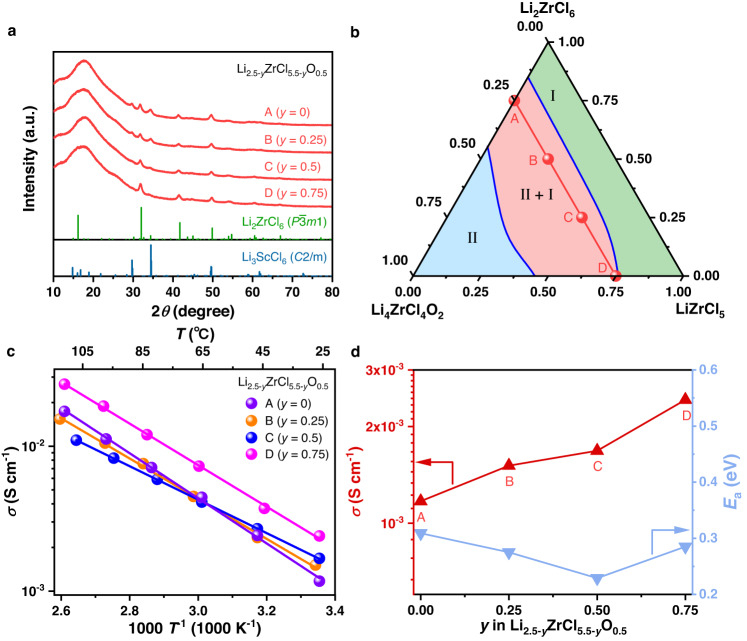
Structures and ionic conductivities of Li_2.5-*y*_ZrCl_5.5-*y*_O_0.5_. **a** XRD patterns of Li_2.5-*y*_ZrCl_5.5-*y*_O_0.5_ with different compositions. No smoothening was conducted to any of the diffraction data displayed here. **b** The Li_2_ZrCl_6_-Li_4_ZrCl_4_O_2_-LiZrCl_5_ ternary phase diagram with compositions A−D in (**a**) indicated by red dots. **c** Arrhenius plots of compositions A−D in (**a**). **d** Ionic conductivities at 25 °C and activation energies of compositions A−D in (**a**).

While the ionic conductivity of Li_2.5_ZrCl_5.5_O_0.5_ already exceeds 1 mS cm^−1^, further improvement is still possible. As shown in Fig. 2b, within the two-phase region, the ionic conductivity also varies; when the compositions are approaching the boundary between Phase I and the two-phase region, the materials would become more ionically conductive. Following this trend, the two-phase compositions that are closer to the aforementioned phase boundary than the most ionically conductive *x* = 0.5 (*a* = 25%) composition identified above might possibly exhibit higher ionic conductivities. Nevertheless, the search of such compositions in Li_2+*x*_ZrCl_6-*x*_O_*x*_ or (1-*a*)Li_2_ZrCl_6_-*a*Li_4_ZrCl_4_O_2_ is challenging, because the difference in the amount of starting materials used for synthesis is too small (< 0.05 g for synthesizing each gram of the material) to be controlled accurately in practice. In light of this issue, we introduced a third component, the LiZrCl_5_ solid electrolyte that is isostructural with Li_2_ZrCl_6_^12^, into (1-*a*)Li_2_ZrCl_6_-*a*Li_4_ZrCl_4_O_2_, and the general formula thereby becomes (1-*a*-*b*)Li_2_ZrCl_6_-*a*Li_4_ZrCl_4_O_2_-*b*LiZrCl_5_ or Li_2+*x*-*y*_ZrCl_6-*x*-*y*_O_*x*_ (*a* = *x*/2, *b* = *y*). Compared with the binary (1-*a*)Li_2_ZrCl_6_-*a*Li_4_ZrCl_4_O_2_ system, the ternary (1-*a*-*b*)Li_2_ZrCl_6_-*a*Li_4_ZrCl_4_O_2_-*b*LiZrCl_5_ materials would likely possess larger room for composition variation within a given phase region, and thus may allow for a more accurate control of the distance between the synthesized compositions and the phase boundary mentioned above. When exploring this ternary system, we fixed *a* at 25% (equivalent to fixing *x* in Li_2+*x*-*y*_ZrCl_6-*x*-*y*_O_*x*_ at 0.5), and let *b* vary between 0 and 75% (equivalent to letting *y* in Li_2+*x*-*y*_ZrCl_6-*x*-*y*_O_*x*_ vary between 0 and 0.75); alternatively, these compositions may also be expressed more briefly as Li_2.5-*y*_ZrCl_5.5-*y*_O_0.5_, where *y* lies between 0 and 0.75. The composition with *y* or *b* equal to 0 corresponds to the most ionically conductive Li_2.5_ZrCl_5.5_O_0.5_ solid electrolyte identified above, and the value of *y* or *b* reflects the content of LiZrCl_5_ introduced into it. Figure 3a displays the XRD patterns of these Li_2.5-*y*_ZrCl_5.5-*y*_O_0.5_ materials. Phase I and Phase II were observed to coexist in all of them. Nevertheless, with the increase of *y*, the Bragg reflections of Phase II, i.e., the one showing the *C2/m* symmetry, gradually weakens. When *y* reaches 0.75, the signals of Phase II are still present, but become barely visible compared to those of Phase I. This observation suggests that the material almost becomes single-phase at *y* = 0.75; among the compositions studied here, it is thereby the closest to the boundary between Phase I and the two-phase region. The phase evolution here can also be illustrated by a Li_2_ZrCl_6_-Li_4_ZrCl_4_O_2_-LiZrCl_5_ ternary phase diagram inferred from the structure-composition relationship disclosed above. As shown in Fig. 3b, since Li_2_ZrCl_6_ is isostructural with LiZrCl_5_, they should belong to the same single-phase region (Phase I). The other end member Li_4_ZrCl_4_O_2_ exhibits a different crystal structure from these two, and thus should reside in another single-phase region (Phase II). Between these two single-phase regions is a two-phase one, where Phase I and Phase II coexist. Since *a* is fixed at 25% for all the (1-*a*-*b*)Li_2_ZrCl_6_-*a*Li_4_ZrCl_4_O_2_-*b*LiZrCl_5_ compositions studied in Fig. 3a, they should be on a line parallel with the Li_2_ZrCl_6_-LiZrCl_5_ edge in the ternary phase diagram; in Fig. 3b, this line is highlighted in red, while points A, B, C, and D on it correspond to the compositions with *b* (equal to *y* in Li_2.5-*y*_ZrCl_5.5-*y*_O_0.5_) being 0, 25%, 50%, and 75%, respectively, i.e., the ones that have been studied by XRD in Fig. 3a. In this way, it can be seen from Fig. 3b that the increase of *b* or *y* from 0 to 75% is essentially moving the compositions closer to the boundary between Phase I and the two-phase region; the one with *y* or *b* equal to75%, i.e., point D in Fig. 3b, is almost single-phase (Fig. 3a), and thus may be considered to lie on the phase boundary. If the closeness to this phase boundary were indeed associated with improved ionic conductivity as speculated above, point D should be the most ionically conductive composition. To verify this hypothesis, conductivity measurements were carried out for compositions A−D in Fig. 3b, i.e., Li_2.5-*y*_ZrCl_5.5-*y*_O_0.5_ with *y* = 0−0.75. All these materials were found to possess ionic conductivities above 1 mS cm^−1^ and negligibly low electronic conductivities of 10^−10^−10^−9^ S cm^−1^ at 25 °C (Supplementary Fig. 14). As expected, the Li-ion transport at 25 °C becomes more and more efficient from points A to D (Fig. 3c, d), and the optimal ionic conductivity at 25 °C (achieved at point D) is as high as 2.42 mS cm^−1^. Such an ionic conductivity improvement should most likely arise from the structural change of the amorphous species, rather than that of the crystalline phases. As reflected by the XRD patterns (Fig. 3a), all the compositions here are as amorphous as composition A, whose crystallinity, i.e., the mass fraction of the crystalline phases, is below 20% (Fig. 1d). With the amount of the amorphous species exceeding that of the crystalline phases, the structural change of the latter is unlikely to cause considerable change in the overall ionic conductivity, so the improvement should arise at least mostly from that of the former. Unfortunately, the atomic configuration of the amorphous species is too complicated to be precisely studied by the present experimental or computational techniques, making it very difficult to conduct in-depth discussion on the microscopic origin of the ionic conductivity improvement associated with the LiZrCl_5_ addition. Regardless, compared with the unmodified Li_2_ZrCl_6_ (ionic conductivity at 30 °C reported as 0.3−0.5 mS cm^−1^ in literature^15^), the materials design presented above does increase the ionic conductivity by nearly one order of magnitude, reaching 2.42 mS cm^−1^ at 25 °C in Li_1.75_ZrCl_4.75_O_0.5_ (composition D in Fig. 3). This composition will be the focus for the rest of the study, and will be abbreviated as LZCO below.

### Compressibility of LZCO

**Fig. 4:**
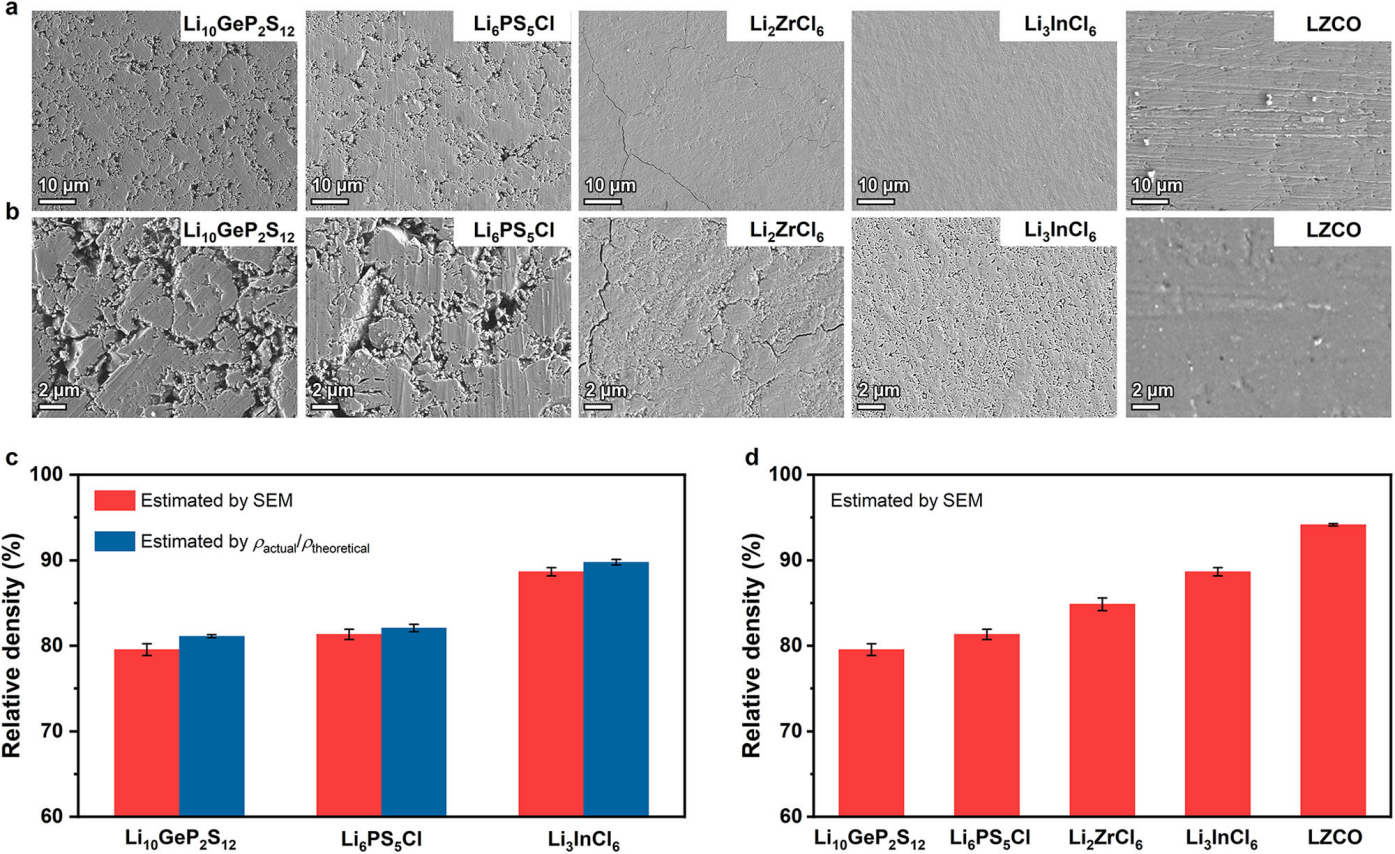
Compressibility of LZCO. **a**−**b** SEM images with low (**a**) and high magnifications (**b**) for the cold-pressed pellets of Li_10_GeP_2_S_12_, Li_6_PS_5_Cl, Li_2_ZrCl_6_, Li_3_InCl_6_, and LZCO. All the pellets were fabricated under 300 MPa. The scale bars in (**a** and **b**) are 10 and 2 μm, respectively. **c** Comparison between the relative densities estimated by SEM and those estimated by comparing the actual density *ρ*_actual_ and theoretical density *ρ*_theoretical_. **d** Relative densities for the five solid electrolytes in (**a** and **b**), all of which were estimated from SEM. Each error bar was determined from the standard deviation of the data from five samples.

The appropriate solid electrolyte for ASSLBs not only needs to be highly ionically conductive, but also must be easily compressible under pressure^7,8^; otherwise an intimate, thorough solid-solid contact would be very difficult to form between the solid electrolyte and electrode’s active material. The compressibility of solid electrolytes can be straightforwardly reflected by the relative density of cold-pressed pellets under a given pressure^25^; the pellets fabricated from more compressible material would exhibit higher relative density. In order to study this characteristic for LZCO, we pressed its powder into a pellet under 300 MPa. For comparison, such cold-pressed pellets were also prepared under the same pressure from four other widely studied solid electrolytes: Li_10_GeP_2_S_12_, Li_6_PS_5_Cl, Li_2_ZrCl_6_, and Li_3_InCl_6_. Unlike LZCO and Li_2_ZrCl_6_, Li_3_InCl_6_ is more ionically conductive in the highly crystalline state, so the planetary mill that is intense enough to decrease the crystallinity will compromise its ionic conductivity^43^. In addition, Li_10_GeP_2_S_12_ and Li_6_PS_5_Cl were found to show the same behavior. When planetary milled using the conditions for synthesizing LZCO, Li_10_GeP_2_S_12_ becomes completely amorphous, and the ionic conductivity is lowered by nearly two orders of magnitude (Supplementary Fig. 15). After such treatment, Li_6_PS_5_Cl also undergoes a drastic decrease in the crystallinity and ionic conductivity (Supplementary Fig. 16). Consequently, for Li_10_GeP_2_S_12_, Li_6_PS_5_Cl, and Li_3_InCl_6_, their planetary milled, low-crystallinity powder would not be used in practical application due to the lower ionic conductivity. Since the comparison here aims at providing a guidance for selecting appropriate solid electrolytes for the practical all-solid-state cells, the three materials mentioned above were not planetary milled like LZCO and Li_2_ZrCl_6_, to ensure that all five of them are in the most ionically conductive state during the comparison. The surface morphologies of these pellets were first examined by scanning electron microscopy (SEM). The images taken under two different magnifications are displayed in Fig. 4a and b, respectively. The pellets of the two sulfide solid electrolytes, i.e., Li_10_GeP_2_S_12_ and Li_6_PS_5_Cl, were found least dense; the large pores (averagely 0.214 and 0.208 μm^2^ for Li_10_GeP_2_S_12_ and Li_6_PS_5_Cl, respectively) are clearly visible in the low-magnification images shown in Fig. 4a. In contrast, the pellets of the two chloride solid electrolytes, i.e., Li_2_ZrCl_6_ and Li_3_InCl_6_, are much denser; their pores are too small (averagely 0.072 and 0.065 μm^2^ for Li_2_ZrCl_6_ and Li_3_InCl_6_, respectively) to be visualized clearly in Fig. 4a, which entails higher compressibility than that of Li_10_GeP_2_S_12_ and Li_6_PS_5_Cl. Regardless, the high-magnification images in Fig. 4b still disclose the existence of numerous pores. Besides, unlike the Li_3_InCl_6_ pellet, the one made from Li_2_ZrCl_6_ shows several cracks at the surface. In contrast, the LZCO pellet prepared using the same condition is almost fully densified; under both magnifications discussed above, the pores are barely visible, and cracks were not observed either (Fig. 4a and b). In addition to the surface morphologies, the cross-sectional images of these cold-pressed pellets also suggest that LZCO is more compressible: among all of the five solid electrolytes compared here, only LZCO shows a homogeneous cross section that is almost free of cracks and pores (Supplementary Fig. 17). According to these results, LZCO should be the most compressible solid electrolyte among the ones examined here.

In order to compare the compressibility in a more quantitative manner, the relative densities of the cold-pressed pellets were estimated. Conventionally, the relative density is determined by comparing the actual and theoretical densities^30,31^. The former density can be measured directly from the pellet, whereas the latter needs to be calculated from the unit-cell configuration determined by XRD. That is, the theoretical density may only be estimated for crystalline species. If non-negligible amounts of amorphous species coexist with the crystalline ones in the material, it would be difficult to know the overall theoretical density, and the absence of such knowledge prevents the accurate determination of the relative density using the aforementioned method. Among the five solid electrolytes examined here, Li_2_ZrCl_6_ and LZCO are both the partially amorphous materials mentioned above; as shown in Fig. 1d, their crystallinities are below 60%, meaning over 40% of the mass are amorphous. As a result, the relative density needs to be determined by alternative approaches. In fact, SEM is a rather straightforward and effective tool for this purpose. In the present study, the porosity of the cold-pressed pellets was first estimated from around 30 SEM images at different regions of the sample using the Adjust-Threshold plugin of ImageJ^44^. Subtracting the average porosity of these SEM images from 100% would yield the relative density. In order to verify the reliability of this approach, it was first used to study Li_10_GeP_2_S_12_, Li_6_PS_5_Cl, and Li_3_InCl_6_, whose high crystallinities allow their relative densities to be determined by comparing the actual and theoretical densities as well^7,43,45^. As shown in Fig. 4c, the relative densities measured by the conventional method are almost identical with those by SEM for all the three materials. With its effectiveness confirmed, SEM was used to compared the relative densities of the cold-pressed pellets made from different solid electrolytes, and the results are displayed in Fig. 4d. Consistent with the images in Fig. 4a and b, the two chloride pellets are denser than the two sulfide ones. Among these four pellets, the one with the highest relative density is the Li_3_InCl_6_ pellet, which is 88.7% dense. In contrast, the LZCO pellet fabricated under the same pressure is 94.2% dense. Therefore, this oxychloride is more compressible than the representative sulfide and chloride solid electrolytes studied here, and also very well satisfies the requirement specified in Introduction, i.e., compressible enough to make the relative density exceed 90% under 250−350 MPa.

### Electrochemical characterizations of the solid-state electrolytes

With the high ionic conductivity and excellent compressibility achieved above, LZCO is expected to enable effective cycling performances in all-solid-state cells. In order to determine an appropriate configuration for the LZCO-based cells, its electrochemical stability was first studied through linear sweep voltammetry (LSV). The cell assembled for this purpose utilizes the mixture of LZCO and C (carbon black) at a weight ratio of 7:3 as the working electrode, LZCO as the solid electrolyte, and Li metal as the counter/reference electrode; to prevent the possible reaction between LZCO and Li metal, they were separated by a thin layer of Li_6_PS_5_Cl (315 μm thick, abbreviated as LPSCl below). For comparison, a similar cell with LZCO replaced by Li_2_ZrCl_6_ (abbreviated as LZC below) was also tested. Supplementary Fig. 18 shows the LSV results for the Li | LPSCl-LZCO | LZCO + C and the Li | LPSCl-LZC | LZC + C cells described above. The reduction onset of LZCO was found lower than that of LZC (1.79 and 2.16 V vs. Li/Li^+^, respectively), even though the reduction of both materials occurs through the same cation, Zr^4+^. Such a difference could arise from two factors. First of all, the potential where a given cation is reduced is in fact dependent on the anions it bonds with. In oxides such as Li_7_La_3_Zr_2_O_12_ and LiZr_2_(PO_4_)_3_, the reduction potential of Zr^4+^ is usually so close to 0 V vs. Li/Li^+^ that the overly weak thermodynamic driving force barely allows the materials to react with Li metal in practice^46,47^. However, in chlorides, the same cation will be reduced at much higher potentials that typically lie around 2 V vs. Li/Li^+^^12,48^. Unlike LZC, the Zr^4+^ in LZCO is bonded not only to Cl^−^, but also to O^2−^; as mentioned above, bonding with O^2−^ would generally make Zr^4+^ display lower reduction potentials, so it appears reasonable to observe that the Zr^4+^ in LZCO is reduced at a slightly lower potential than that in LZC. Secondly, whether the material is amorphous or crystalline could also significantly influence the reduction potential. For example, the difference between the reduction potentials of the amorphous and crystalline Li_0.35_La_0.55_TiO_3_ is reported to be as large as 0.5 V^49^. For LZCO, the majority of the material is amorphous, so the LSV signals from its crystalline phases should be very weak, or non-detectable. In contrast, the high crystallinity of LZC (Fig. 1d) suggests that its crystalline phase would contribute non-negligible signals during the LSV measurement. Such a difference could also result in the distinction between the reduction potentials of LZCO and LZC. Although the LZCO material reported here shows better reduction stability than LZC, such an improvement is still not sufficient to enable a compatibility with the Li metal negative electrode. As shown in Supplementary Fig. 19, the Li | LZCO | Li symmetric cell shows an increase in the Li stripping/plating voltages during cycling; although the voltages eventually stabilized, the overpotential was still too high. Therefore, the Li metal negative electrode should not be in direct contact with LZCO in the cell. On the other hand, the oxidation onset of LZCO was measured to be 4.0 V vs. Li/Li^+^, much higher than that of LZC (3.55 V vs. Li/Li^+^). It should be noted that the chloride solid electrolytes can typically enable stable cell cycling to voltages beyond their own oxidation potentials. For example, the Li_2_Sc_2/3_Cl_4_ solid electrolyte shows an oxidation potential of only 4.3 V vs. Li/Li^+^, but the uncoated LiNi_0.6_Mn_0.2_Co_0.2_O_2_ in direct contact with Li_2_Sc_2/3_Cl_4_ can be stably cycled to a higher voltage of 4.6 V vs. Li/Li^+^^50^. The Li_2_ZrCl_6_ solid electrolyte will be oxidized at a rather low potential of around 3.5 V vs. Li/Li^+^, but it does allow the uncoated LiNi_0.88_Co_0.11_Al_0.01_O_2_ and LiNi_0.8_Mn_0.1_Co_0.1_O_2_ to be cycled stably to 4.3 and 4.4 V vs. Li/Li^+^, respectively^12,15^. It has been speculated that the chloride solid electrolytes will still react with the 4 V-class positive electrodes, but the resulting interphase can both prevent further reaction and enable relatively efficient ion transport^50^. Since the O content in LZCO is rather low in comparison with that of Cl (Cl:O molar ratio is 4.75:0.5), it is unlikely to fundamentally alter the aforementioned interfacial behavior. As a result, it appears reasonable to believe that LZCO will inherit such a desirable characteristic, i.e., the capability of enabling stable cycling to voltages beyond its own oxidation potential, from the chloride solid electrolytes. In this way, with an oxidation potential of 4.0 V vs. Li/Li^+^, LZCO should supposedly allow the uncoated 4 V-class positive electrodes to be stably cycled at higher voltages. As a matter of fact, the cycling tests of all-solid-state cells below do suggest this hypothesis is correct.

**Fig. 5:**
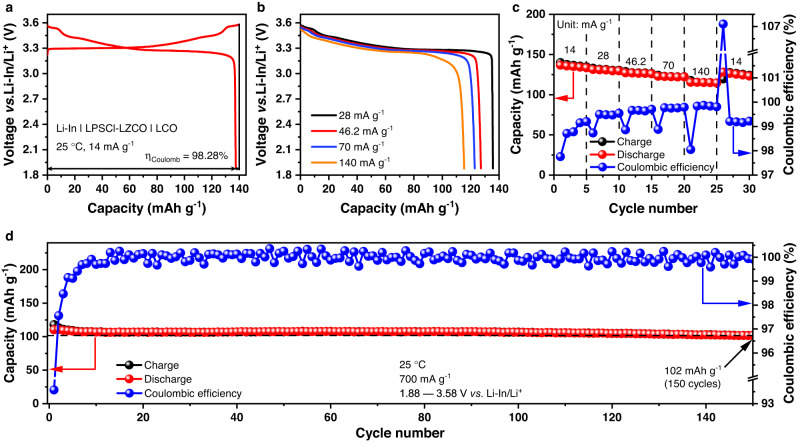
Electrochemical performance of the Li-In | LPSCl-LZCO | LCO cell. **a** Initial charge and discharge voltage profiles at 14 mA g^–1^, with the Coulombic efficiency *η*_Coulomb_ denoted. **b**, **c** Rate capability at 14, 28, 46.2, 70, and 140 mA g^–1^. **d** Long-term cycling performance at 700 mA g^–1^. All the cycling tests were conducted between 1.88 and 3.58 V vs. Li-In/Li^+^ at 25 °C and 1.5 tons of applied external pressure.

According to the ESW determined above, all-solid-state cells were assembled using LiCoO_2_ (LCO) or single-crystalline LiNi_0.8_Mn_0.1_Co_0.1_O_2_ (scNMC811) particles without any extra particle coating as the positive electrode active materials, LZCO as the solid electrolyte, and Li-In alloy as the negative electrode; considering that the LZCO solid electrolyte with a reduction potential of 1.79 V vs. Li/Li^+^ will react with the Li-In alloy negative electrode, they were separated by a thin layer of LPSCl (220 μm thick). Figure 5a shows the initial charge/discharge voltage profiles of the Li-In | LPSCl-LZCO | LCO cell at 14 mA g^–1^ between 1.88 and 3.58 V vs. Li-In/Li^+^ at 25 °C and 1.5 tons of applied external pressure; it delivered an initial discharge capacity of 137.5 mAh g^−1^ (based on the mass of the active material in the positive electrode) and a Coulombic efficiency of 98.28%, which surpass those of most LCO-based all-solid-state cells in literature^9,35,43^. Furthermore, the Li-In | LPSCl-LZCO | LCO cell also exhibits good rate capability (Fig. 5b−c). As the specific current increased stepwise from 14 to 140 mA g^–1^, the capacity fade upon each rate change is barely visible in the data plot (Fig. 5c). Besides, when the initial cycle was run at specific currents higher than the 14 mA g^–1^ one used in Fig. 5a, the cell performance does not decay considerably either; at 70, 140, 420 and 700 mA g^–1^, the initial discharge capacities of 129.7, 126.6, 118.9, and 109.7 mAh g^−1^ with Coulombic efficiencies of 98.31%, 97.67%, 95.47%, and 94.33%, respectively, may still be achieved (Supplementary Fig. 20). Such performance suggests that the long-term cycling of the Li-In | LPSCl-LZCO | LCO cell might be conducted at the specific currents higher than those usually adopted in literature. At present, 10−30 mA g^–1^ are the most commonly applied specific currents for demonstrating the cycling stability of all-solid-state cells based on LiCoO_2_ and LiNi_0.8_Mn_0.1_Co_0.1_O_2_^9,51,52^. Cycling at higher specific currents like 150−200 mA g^–1^ are much less frequently conducted^12,50^; recently, a Li_2_In_1/3_Sc_1/3_Cl_4_ solid electrolyte with good cycling performance was reported to enable long-term cycling at a specific current as high as 540 mA g^–1 ^^53^. In the present study, an even higher specific current of 700 mA g^–1^ was adopted for the long-term cycling. The result of the Li-In | LPSCl-LZCO | LCO cell is displayed in Fig. 5d and Supplementary Fig. 21. Except for a slight decrease in the first several cycles, the capacity remained largely unchanged for 150 cycles. At the end of such cycling, the final discharge capacity maintained at 102 mAh g^−1^, with a Coulombic efficiency of 99.90%. Interestingly, if LZCO was replaced by another high-performance solid electrolyte LZC in the cell, and the cycling was performed at a much lower specific current of 70 mA g^–1^ (as in one of our recent studies^12^), the discharge capacity after 100 cycles was only 114 mAh g^–1^, not much different from that achieved above under 700 mA g^–1^. This fact further demonstrates the good cycling performance of the Li-In | LPSCl-LZCO | LCO cell. Beyond the cycling conditions explored above, the cell can also deliver satisfactory performance at higher upper cutoff voltages. When being cycled between 1.88 and 3.68 V vs. Li-In/Li^+^ at 25 °C and 1.5 tons of applied external pressure, the Li-In | LPSCl-LZCO | LCO cell showed an initial discharge capacity of 145.6 mAh g^−1^ with a Coulombic efficiency of 97.73% under 79 mA g^–1^ at 25 °C (Supplementary Fig. 22a), and a decent rate performance was achieved as well (Supplementary Fig. 22b−c). At the specific current of 790 mA g^–1^, the cell maintained a discharge capacity of 104.1 mAh g^−1^ with a Coulombic efficiency of 99.90% after 200 cycles (Supplementary Fig. 22d). When the upper cutoff voltage was further increased to 3.78 V vs. Li-In/Li^+^, an initial discharge capacity of 151.1 mAh g^−1^ with a Coulombic efficiency of 97.56% was achieved under 86 mA g^–1^ at 25 °C (Supplementary Fig. 23a). In addition to the fine rate performance (Supplementary Fig. 23b−c), a good cycling stability was observed as well: after 400 cycles under 860 mA g^–1^ at 25 °C, a discharge capacity of 100.6 mAh g^−1^ with a Coulombic efficiency of 99.92% can still be delivered (Supplementary Fig. 23d).

**Fig. 6:**
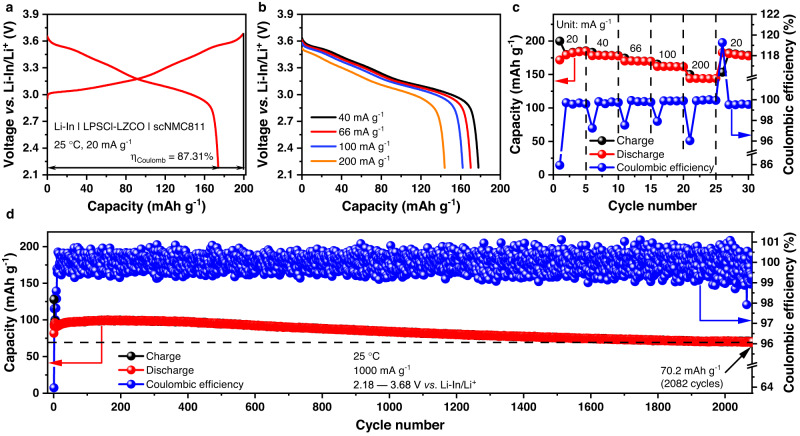
Electrochemical performance of the Li-In | LPSCl-LZCO | scNMC811 cell. **a** Initial charge and discharge voltage profiles at 20 mA g^–1^, with the Coulombic efficiency *η*_Coulomb_ denoted. **b**, **c** Rate capability at 20, 40, 66, 100, and 200 mA g^–1^. **d** Long-term cycling performance at 1000 mA g^–1^. All the cycling tests were conducted between 2.18 and 3.68 V vs. Li-In/Li^+^ at 25 °C and 1.5 tons of applied external pressure.

Beyond the LCO positive electrode, LZCO also enabled good battery performances for scNMC811. As shown in Fig. 6a, when the Li-In | LPSCl-LZCO | scNMC811 cell was cycled at 20 mA g^–1^ between 2.18 and 3.68 V vs. Li-In/Li^+^ at 25 °C and 1.5 tons of applied external pressure, it delivered an initial discharge capacity of 173.96 mAh g^–1^ with a Coulombic efficiency of 87.31%. As the specific current increased stepwise from 20 to 200 mA g^–1^, a rate performance similar to that observed for the Li-In | LPSCl-LZCO | LCO cell above was achieved too (Fig. 6b−c). Most importantly, the Li-In | LPSCl-LZCO | scNMC811 cell also shows good long-term cycling stability at 1000 mA g^–1^ (Fig. 6d and Supplementary Fig. 24). The capacity barely faded for nearly 500 cycles. After 2082 cycles, a discharge capacity of 70.2 mAh g^–1^ and Coulombic efficiency of 99.95% were still observed. In addition to the voltage range of 2.18 and 3.68 V vs. Li-In/Li^+^ studied above, the Li-In | LPSCl-LZCO | scNMC811 cell was cycled with a higher upper cutoff cell voltage of 3.78 V vs. Li-In/Li^+^. The initial charge and discharge curves of the cell cycled between 2.18 and 3.78 V vs. Li-In/Li^+^ under 20 mA g^–1^ at 25 °C are displayed in Supplementary Fig. 25a; the data indicate a high initial capacity of 175.56 mAh g^–1^ with Coulombic efficiency of 87.83%. Besides, the cycling stability is also satisfactory. As shown in Supplementary Fig. 25b−c, the cell maintained a discharge capacity of 99.9 mAh g^−1^ with a Coulombic efficiency of 99.94% after 570 cycles under 600 mA g^−1^ at 25 °C. According to the data summarized recently by Zhou et al.^53^, the performances of the LZCO-based cells presented above are comparable with the best ones reported for ASSLBs in literature.

### Cost-effectiveness of LZCO

The electrochemical energy storage performances presented above are not realized at the expense of economical affordability; on the contrary, LZCO is highly cost-competitive. In order to realize successful commercialization for ASSLBs, the material cost of solid electrolytes must not exceed $50/kg^27,28^. Nevertheless, this goal is very difficult to achieve if the requirements on ionic conductivity (above 1 mS cm^−1^ at 25 °C) and compressibility (high enough to realize over 90% density under 250−350 MPa) need to be satisfied too. Among the reported inorganic solid electrolytes, only sulfides and halides can be highly Li-ion conductive and easily compressible at the same time^2,8^, but both are plagued by cost issues^12^. As mentioned in the “Introduction” section, the expensive raw material Li_2_S makes the sulfide solid electrolytes cost no less than $196.25/kg (the cost estimations here and below are all based upon the unit prices of 1000 kg purchase listed in a recent report^12^, and the cost analysis method we used^54^ is described in Supplementary Note 1). As for halides, sufficiently cost-effective systems like Li_2_ZrCl_6_ do exist, but their ionic conductivities are below 1 mS cm^−1^ at 25 °C^12,15^. The halides with higher Li-ion conductivities are still rather expensive, because they contain elements with low abundance in Earth’s curst^8,12^; the representative systems include Li_3_InCl_6_, Li_3_ScCl_6_, and Li_2_In_1/3_Sc_1/3_Cl_4_^8,50^, whose material costs are estimated as $380.26/kg, $9468.84/kg, and $4418.10/kg, respectively. In comparison, LZCO involves neither expensive raw materials like Li_2_S nor low-abundance elements like those in the halides mentioned above. It can be synthesized straightforwardly from LiCl, ZrCl_4_, and Li_2_O, while Li_2_O may be easily prepared from even cheaper chemicals such as Li_2_CO_3_, LiOH, and their hydrates^12^. If LiOH·H_2_O, LiCl, and ZrCl_4_ are considered as the raw materials for LZCO, the material cost would be only $11.60/kg, which is significantly lower than the aforementioned $50/kg threshold for the commercialization of ASSLBs.

### Comparison of the Li_1.75_ZrCl_4.75_O_0.5_ and Li_2_ZrCl_6_ physicochemical and electrochemical properties

Although the Li_1.75_ZrCl_4.75_O_0.5_ (LZCO) material designed here possesses a similar chemical formula with the Li_2_ZrCl_6_ (LZC) solid electrolyte already reported in the literature^12,15^, the improvement achieved is significant. First of all, since LZCO simultaneously exhibits improved ionic conductivity, compressibility, and electrochemical stability, the all-solid-state cells it forms completely outperform those based on LZC. For example, the Li-In | LPSCl-LZCO | LCO cell in Fig. 5 and the Li-In | LPSCl-LZC | LCO cell reported in our earlier study on LZC^12^ shows similar configuration; the only difference is that LZCO and LZC were used in the two cells, respectively. Regardless, even though the LZCO-based cell studied here was cycled at a specific current ten times that reported previously for the LZC-based one (700 vs. 70 mA g^–1^), the former still delivered a capacity rather close to that of the latter after 100 cycles (106.5 vs. 114.0 mAh g^−1^). Such cell performance suggests that the advantage of LZCO over LZC is substantial. Secondly, as pointed out in the “Introduction” section, an effective solid electrolyte for all-solid-state Li-based batteries needs to excel simultaneously in at least three aspects, i.e., the ionic conductivity (> 1 mS cm^−1^ at 25 °C), the compressibility (high enough to realize over 90% density under 250−350 MPa), and the cost-effectiveness (below $50/kg), but no such solid electrolyte has been identified in literature. While LZC fails to meet two of these three requirements, i.e., the conductivity and compressibility, LZCO satisfies all of them: it simultaneously exhibits an ionic conductivity of 2.42 mS cm^−1^ at 25 °C, a compressibility that allows the material to become 94.2% dense under 300 MPa, and an estimated cost of $11.60/kg. Last but not least, the discovery of LZCO demonstrates that the barely explored two-phase compositions in the phase diagram could give rise to higher ionic conductivities than the single-phase ones.

In summary, through the design and synthesis of an oxychloride Li_1.75_ZrCl_4.75_O_0.5_ (LZCO), the present research work demonstrates the achieving of an ideal ionic conductivity, compressibility, and cost-effectiveness for solid electrolytes in ASSLBs. Unlike other widely studied solid electrolytes, LZCO is a material where two crystalline phases coexist in a highly amorphized form; one of them exhibits the $$P\bar{3}m1$$ symmetry like Li_2_ZrCl_6_, and the other shows the *C2/m* symmetry like Li_3_ScCl_6_. In the Li_2_ZrCl_6_-Li_4_ZrCl_4_O_2_-LiZrCl_5_ phase diagram, LZCO may be considered as a phase-boundary composition. It shows an ionic conductivity of 2.42 mS cm^−1^ at 25 °C, which exceeds those reported for most (if not all) of the halide solid electrolytes^8,53^ and is also competitive against the sulfide systems^2,4^. Moreover, LZCO is easily compressible. Under a pressure of 300 MPa, its relative density reaches 94.2%, and surpasses those of several well-known, compressible solid electrolytes, including Li_10_GeP_2_S_12_, Li_6_PS_5_Cl, Li_2_ZrCl_6_, and Li_3_InCl_6_ (all below 90%). The simultaneous achievement of such ionic conductivity and compressibility enables good cycling performances. The all-solid-state Li-based cell utilizing single-crystalline LiNi_0.8_Mn_0.1_Co_0.1_O_2_ operated stably at 25 °C under a high rate of 1000 mA g^–1^, and delivered a discharge capacity of 70.2 mAh g^−1^ after 2082 cycles. Beyond these battery performances, LZCO is also rather cost-competitive. Its material cost is estimated as $11.60/kg, which is not only much lower than those of other compressible solid electrolytes with over 1 mS cm^−1^ ionic conductivity at 25 °C (above $196.25/kg), but is also well below the $50/kg threshold suggested for the possible commercialization of ASSLBs^27,28^.

## Methods

### Materials

The Li_2+*x*-*y*_ZrCl_6-*x*-*y*_O_*x*_ solid electrolytes were synthesized from LiCl (Alfa Aesar, 99.9%), ZrCl_4_ (Acros Organics BVBA, 98%), and Li_2_O (Aladdin, 99.99%). Stoichiometric amounts of the starting materials were first mixed manually in an agate mortar for 10 min within an argon-filled glovebox, where the H_2_O and O_2_ contents are both below 0.01 ppm. The resulting mixture was then milled in argon atmosphere in zirconia pots (80 ml) using zirconia balls (5 mm diameter) with a ball-to-powder mass ratio of 15:1 on a planetary mill (FRITSCH, Pulverisette 7 premium line). The milling was first conducted at 150 rpm for 30 min to further mix the starting materials, and then at a higher speed of 600 rpm for 45 h to yield the final products. The synthesis of Li_3_InCl_6_ began with mixing and milling the stoichiometric amounts of LiCl (Alfa Aesar, 99.9%) and InCl_3_ (Alfa Aesar, 99.99%) with similar procedures as those described above, except that the planetary mill was conducted at 500 rpm for 25 h. Afterwards, the milled powder was sealed in a vacuum quartz tube and annealed at 350 °C for 5 h. The Li_10_GeP_2_S_12_ and Li_6_PS_5_Cl solid electrolytes were purchased from Shenzhen Kejing Star Technology Company.

### Structure and morphology characterizations

The crystal structures were studied through powder X-ray diffraction (XRD) using Cu Kα1 radiation; during the measurement, the samples were sealed in Kapton films to prevent air exposure. The Rietveld refinement was performed using GSAS II^55^. Before the XRD patterns used for Rietveld refinement were collected, the samples were mixed with Ag powder (Macklin, 99.5%, 60−120 nm), which serves two purposes. On the one hand, the Bragg reflections of Ag provide the internal standard that ensures an accurate determination of peak positions and lattice parameters. On the other hand, the determination of crystallinity through the method proposed by Yasukawa et al. also needs to use the amount of Ag powder as a reference^39^. The XRD patterns used for Rietveld refinement were collected at 3 degrees per minute, while others were collected at 5 degrees per minute. The surface morphologies of the cold-pressed pellets were observed using a ZEISS GeminiSEM 500 scanning electron microscope operated at 3 kV. The porosity and the size of the pores were estimated from SEM images by using the Adjust-Threshold plugin of ImageJ^44^.

### Conductivity measurements

Prior to the conductivity measurement, around 150 mg of the solid electrolyte powder was first placed in a polyetheretherketone (PEEK) mold with 10 mm diameter in an Ar-filled glovebox with both the H_2_O and O_2_ contents below 0.01 ppm, and then pressed between two stainless steel rods (Anhui Keguan Electronic Technology Company, 17-4PH) at 2.5 tons for 3 min using a hydraulic press (Hefei Kejing Star Technology Company, YLJ-40TA-PE). Afterwards, the pressure was increased to 2.8 tons and maintained at this value during measurement. The ionic conductivity was determined by the electrochemical impedance spectroscopy (EIS) measurement, which was conducted with 10 mV driving potential amplitude from 35 MHz to 1 Hz using a MTZ-35 impedance analyzer (Bio-Logic). The electronic conductivity was determined by the direct current (DC) polarization measurement with the applied voltage of 1 V.

### Electrochemical measurements

The electrochemical stabilities of LZCO and LZC were evaluated through the linear sweep voltammetry (LSV) measurements on the Li | LPSCl-LZCO | LZCO + C and Li | LPSCl-LZC | LZC + C cells, respectively. First of all, 70 mg of LZCO or LZC powder was pressed in a PEEK mold with 10 mm diameter under a pressure of 1.5 tons. Subsequently, 10 mg of working electrode (prepared by manually mixing LZCO or LZC with C (carbon black, Shenzhen Kejing Star Technology Company, 99.9%, 20 nm) at a weight ratio of 7:3) was uniformly sprinkled on one side of the LZCO or LZC pellet, and pressed under 2.4 tons. On the other side of the LZCO or LZC pellet, 50 mg of LPSCl powder (Shenzhen Kejing Star Technology, 99%) was pressed at 1.5 tons, and then a piece of Li foil (200 μm thick, 99.9%, Tianjin Energy Lithium Co. LTD) was attached to serve as the counter electrode. The LSV measurements were performed using CH Instruments CHI630E electrochemical analyzer with a scan rate of 0.1 mV s^−1^ at 25 °C. All the procedures were conducted in an Ar-filled glovebox with both the H_2_O and O_2_ contents below 0.01 ppm.

The all-solid-state cells used for cycling tests were fabricated in the Ar-filled glovebox with both the H_2_O and O_2_ contents below 0.01 ppm as well. The composite positive electrode was prepared by mixing LiCoO_2_ (Alfa Aesar, 99.5%) or single-cystal LiNi_0.8_Mn_0.1_Co_0.1_O_2_ (Hunan Shanshan Energy Technology, 99.9%) with LZCO at a weight ratio of 75:25 using a vortex mixer (Haimen Kylin-Bell Lab Instruments, QL-866) at 1500 rpm for 12 min. The cell assembly began with pressing 40 mg of LZCO powder in a PEEK mold with 10 mm diameter at 1.5 tons for 1 min using a hydraulic press (Hefei Kejing Star Technology Company, YLJ-40TA-PE). Subsequently, the composite positive electrode corresponding to the active material mass loading of 5−6 mg cm^−2^ was dispersed on one side of the cold-pressed LZCO pellet and pressed at 2.4 tons for 3 min. To avoid reaction between LZCO and the negative electrode, 35 mg of LPSCl powder (Shenzhen Kejing Star Technology, 99%) was dispersed evenly on the other side of the LZCO layer, and then pressed at 1.5 tons for 1 min. Finally, a piece of indium foil (Alfa Aesar, 99.99%, 10 mm diameter, 0.1 mm thickness) was placed on top of the LPSCl layer, followed by the attachment of a piece of Li foil (Tianjin Energy Lithium Co. LTD, 99.95%, 10 mm diameter, 50 μm thickness). The cells were cycled under an external pressure of 1.5 tons at 25 °C using a LAND CT2001A battery testing system. The temperature of 25 °C was ensured by placing the cells in an incubator (Tianjin Hongnuo Instrument, SPX-250B, temperature accuracy ± 1 °C) during cycling. In order to ensure the reproducibility of the results, at least ten cells were tested for each electrochemical experiment. The mass of the specific current and that of the specific capacity in the present study refer to the mass of the active material in the positive electrode.

## Supplementary information

Supplementary Information

## Data Availability

The data that support the findings of this study are available within the paper (and its Supplementary Information files) and from the corresponding authors upon reasonable request.
