# Supplementary material for: A cost-effective, ionically conductive and compressible oxychloride solid-state electrolyte for stable all-solid-state lithium-based batteries
Source: Nat Commun. 2023 Jun 27;14:3807. doi: 10.1038/s41467-023-39522-1 (PMC10300059; doi:10.1038/s41467-023-39522-1)
Supplement: Supplementary file 1 — Supplementary Information [file 41467_2023_39522_MOESM1_ESM.pdf]

Supplementary Information

A cost-effective, ionically conductive and compressible oxychloride solid-state electrolyte for stable all-solid-state lithium-based batteries

Lv Hu[1], Jinzhu Wang[1], Kai Wang[1], Zhenqi Gu[1], Zhiwei Xi[1], Hui Li[1], Fang Chen[1], Youxi Wang[2], Zhenyu Li[2] and Cheng Ma[1,3*]

[1]Hefei National Research Center for Physical Sciences at the Microscale, CAS Key Laboratory of Materials for Energy Conversion, Department of Materials Science and Engineering, University of Science and Technology of China, Hefei, Anhui 230026, China.

[2]Key Laboratory of Precision and Intelligent Chemistry, University of Science and Technology of China, Hefei, Anhui 230026, China.

[3]National Synchrotron Radiation Laboratory, Hefei, Anhui 230026, China.

*Corresponding author: mach16@ustc.edu.cn

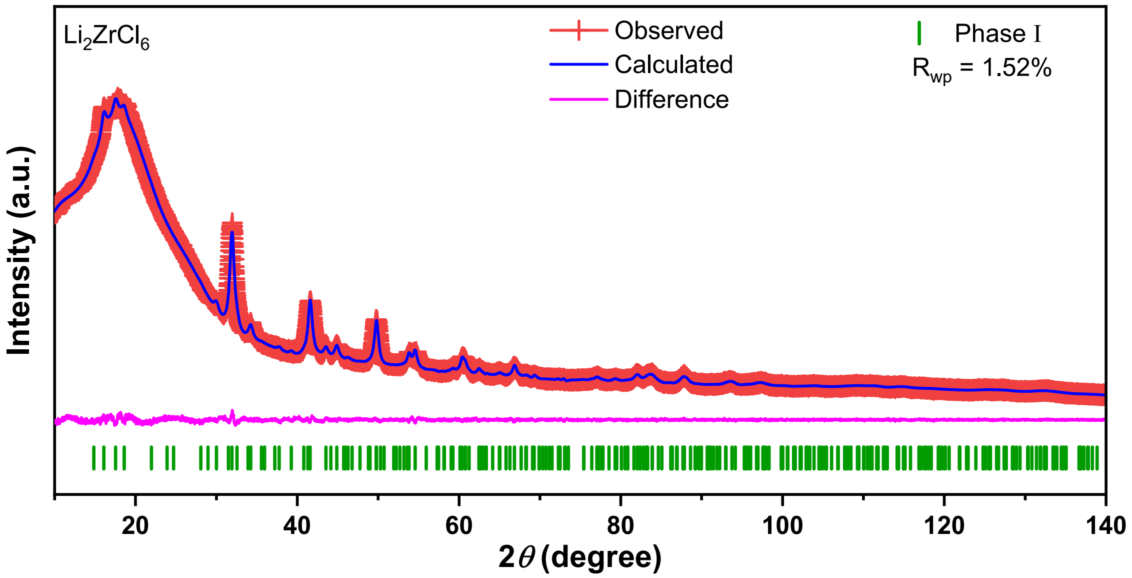

Supplementary Fig. 1. Rietveld refinement of the XRD pattern for Li_2ZrCl_6. No smoothening was conducted to the experimental data used for refinement.

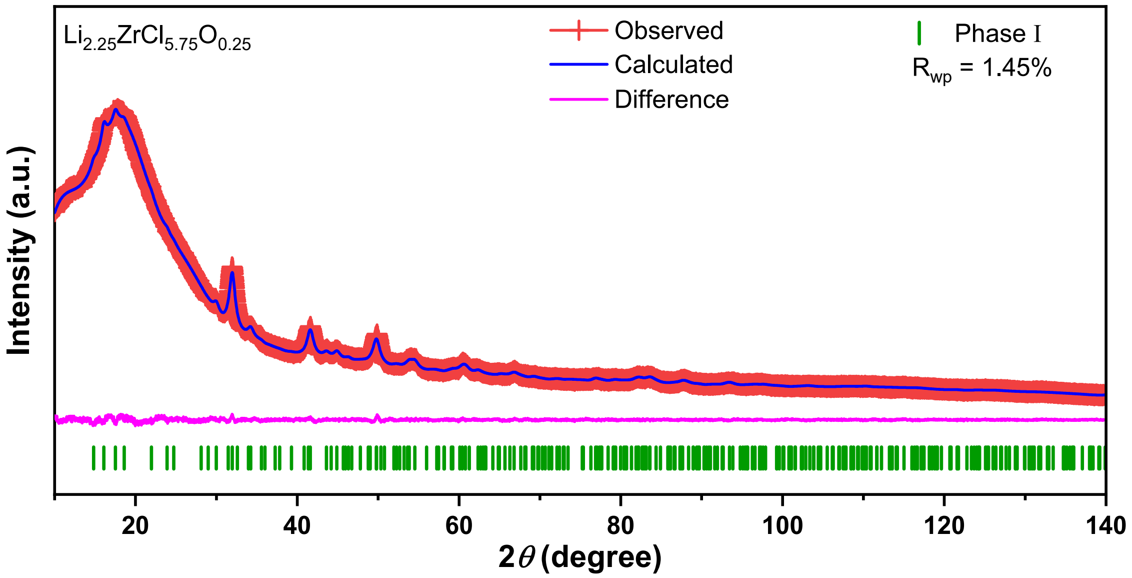

Supplementary Fig. 2. Rietveld refinement of the XRD pattern for $Li_{2.25}ZrCl_{5.75}O_{0.25}$. No smoothening was conducted to the experimental data used for refinement.

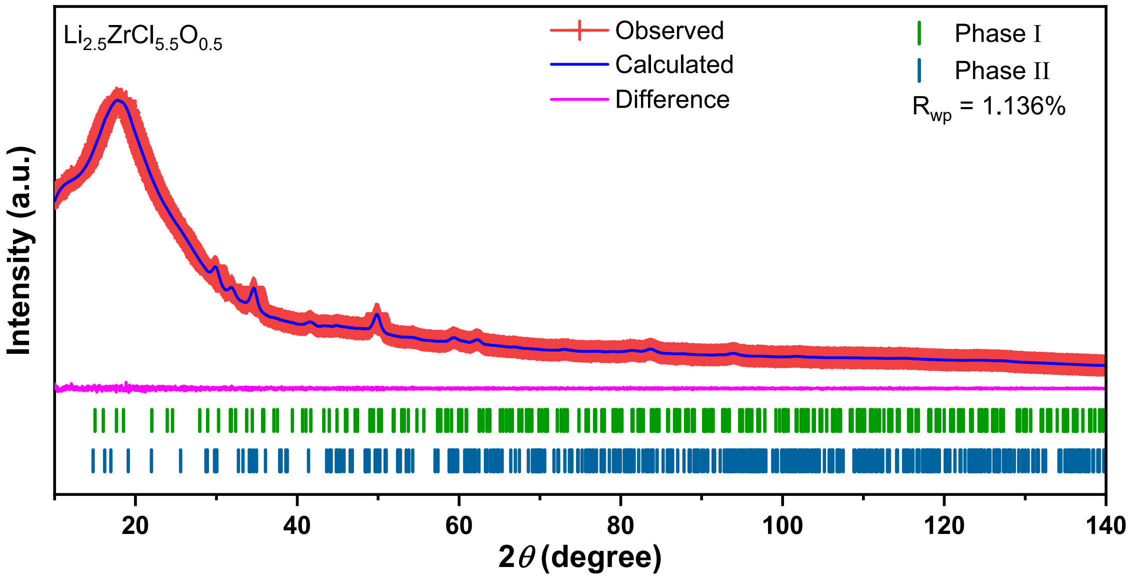

Supplementary Fig. 3. Rietveld refinement of the XRD pattern for $Li_{2.5}ZrCl_{5.5}O_{0.5}$. No smoothening was conducted to the experimental data used for refinement.

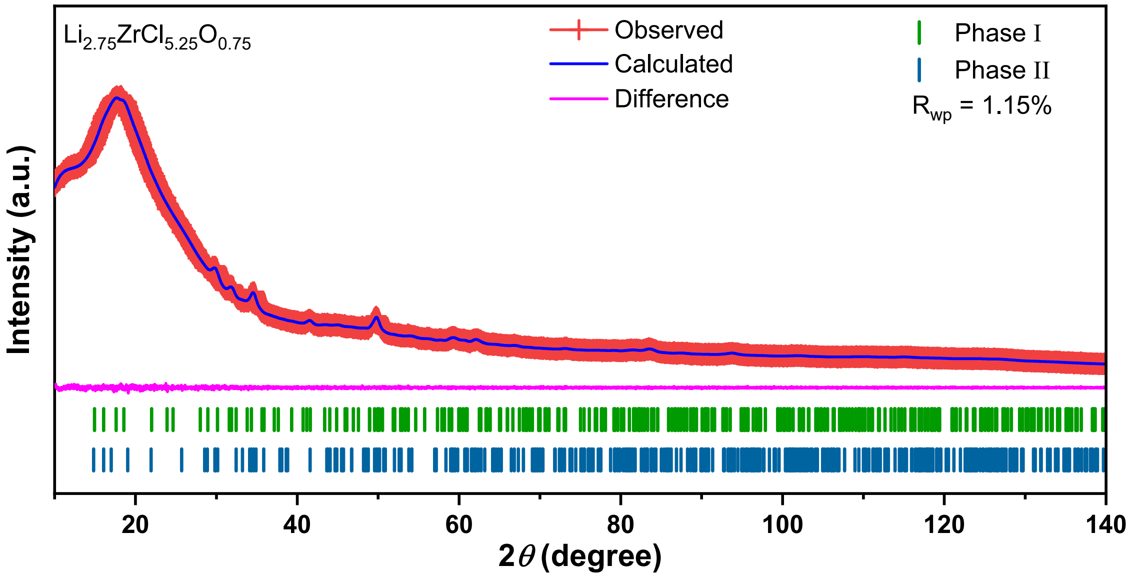

Supplementary Fig. 4. Rietveld refinement of the XRD pattern for $Li_{2.75}ZrCl_{5.25}O_{0.75}$. No smoothening was conducted to the experimental data used for refinement.

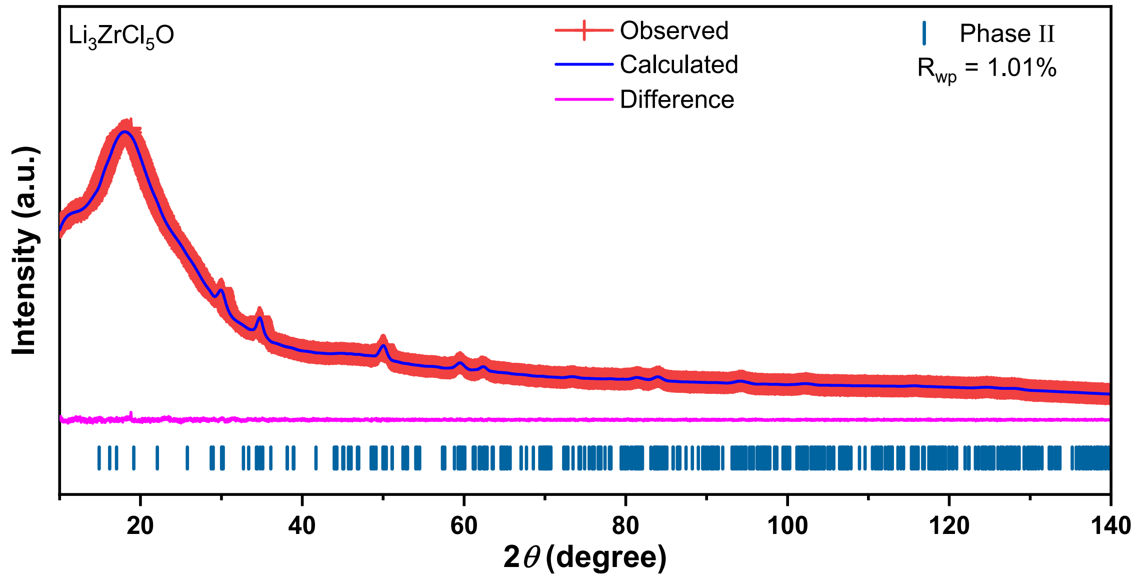

Supplementary Fig. 5. Rietveld refinement of the XRD pattern for Li_3ZrCl_5O. No smoothening was conducted to the experimental data used for refinement.

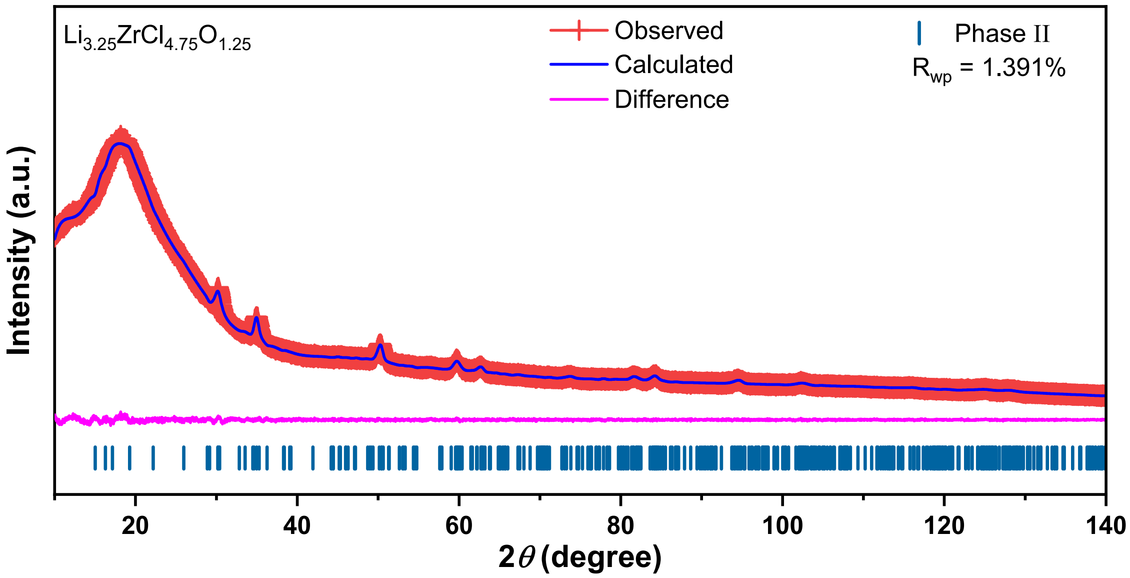

Supplementary Fig. 6. Rietveld refinement of the XRD pattern for $Li_{3.25}ZrCl_{4.75}O_{1.25}$. No smoothening was conducted to the experimental data used for refinement.

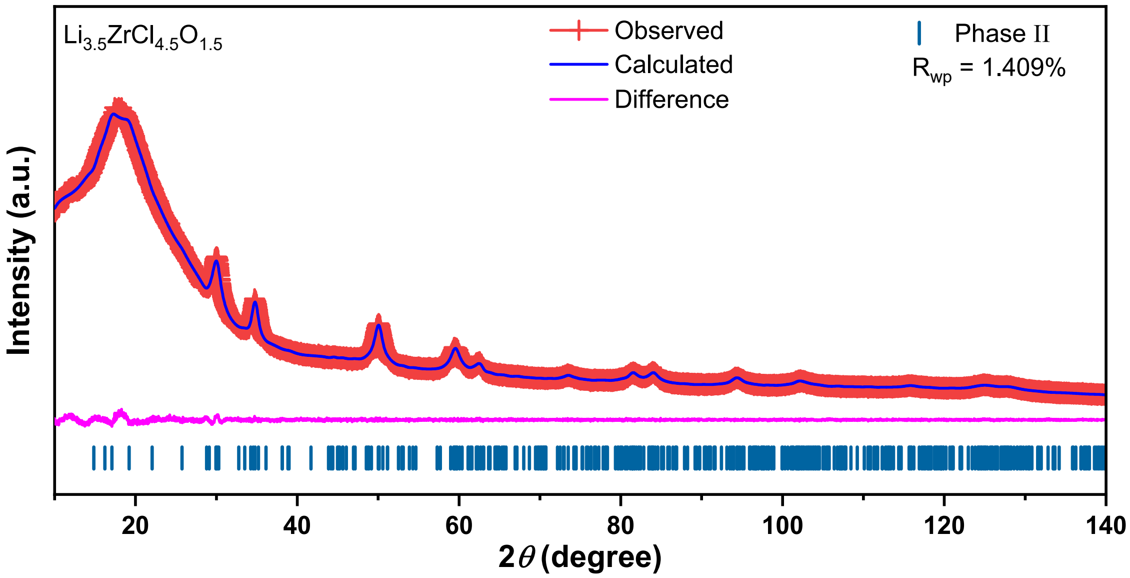

Supplementary Fig. 7. Rietveld refinement of the XRD pattern for $Li_{3.5}ZrCl_{4.5}O_{1.5}$. No smoothening was conducted to the experimental data used for refinement.

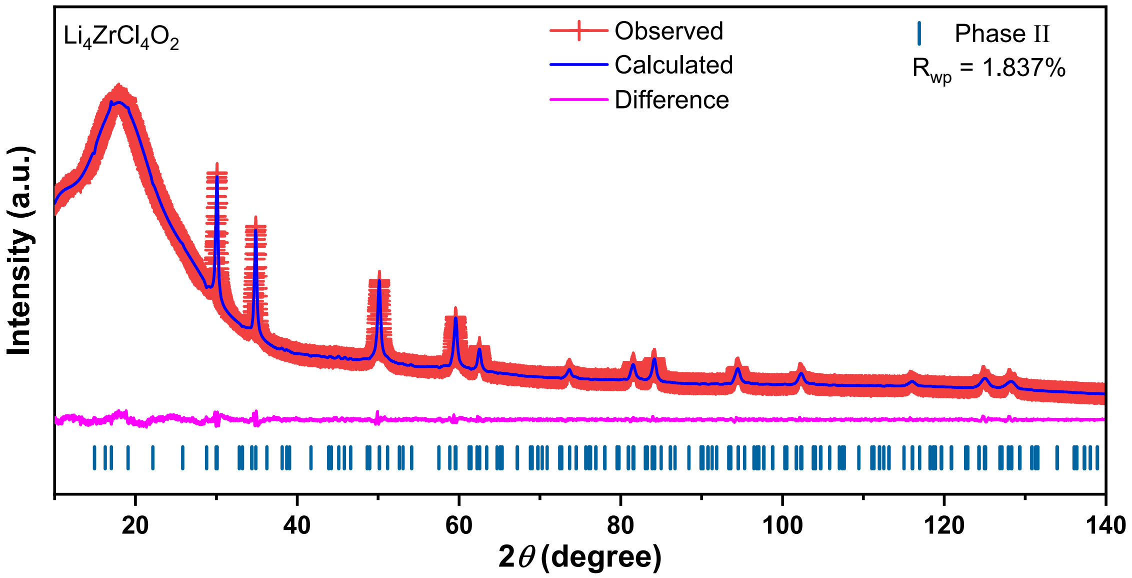

Supplementary Fig. 8. Rietveld refinement of the XRD pattern for $Li_4ZrCl_4O_2$. No smoothening was conducted to the experimental data used for refinement.

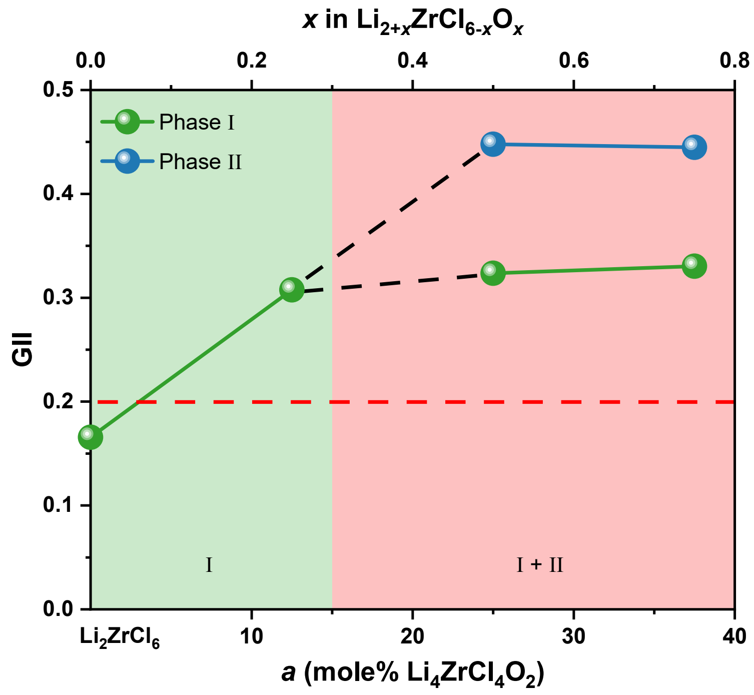

Supplementary Fig. 9. Variation of the GII with the x in $Li_{2+x}ZrCl_{6-x}O_x$.

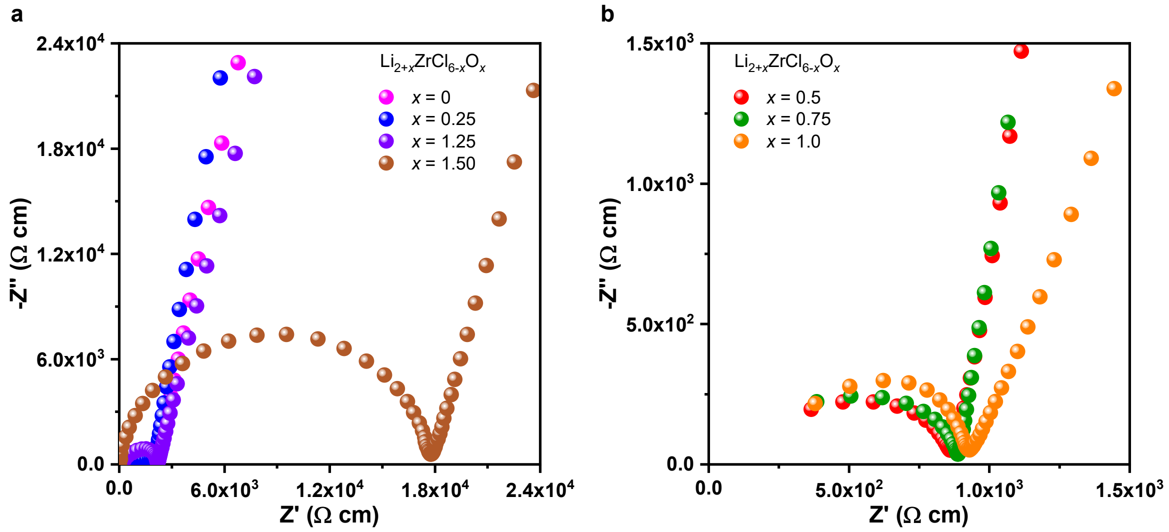

Supplementary Fig. 10. a, Nyquist plots of the $Li_{2+x}ZrCl_{6-x}O_x$ materials with $x = 0$, 0.25, 1.25, and 1.50 at 25 °C. **b**, Nyquist plots of the $Li_{2+x}ZrCl_{6-x}O_x$ materials with $x = 0.5$, 0.75, and 1.0 at 25 °C. The EIS measurement was performed using the cold-pressed pellet with 10 mm diameter sandwiched between two stainless steel electrodes, under an external pressure of 2.8 tons.

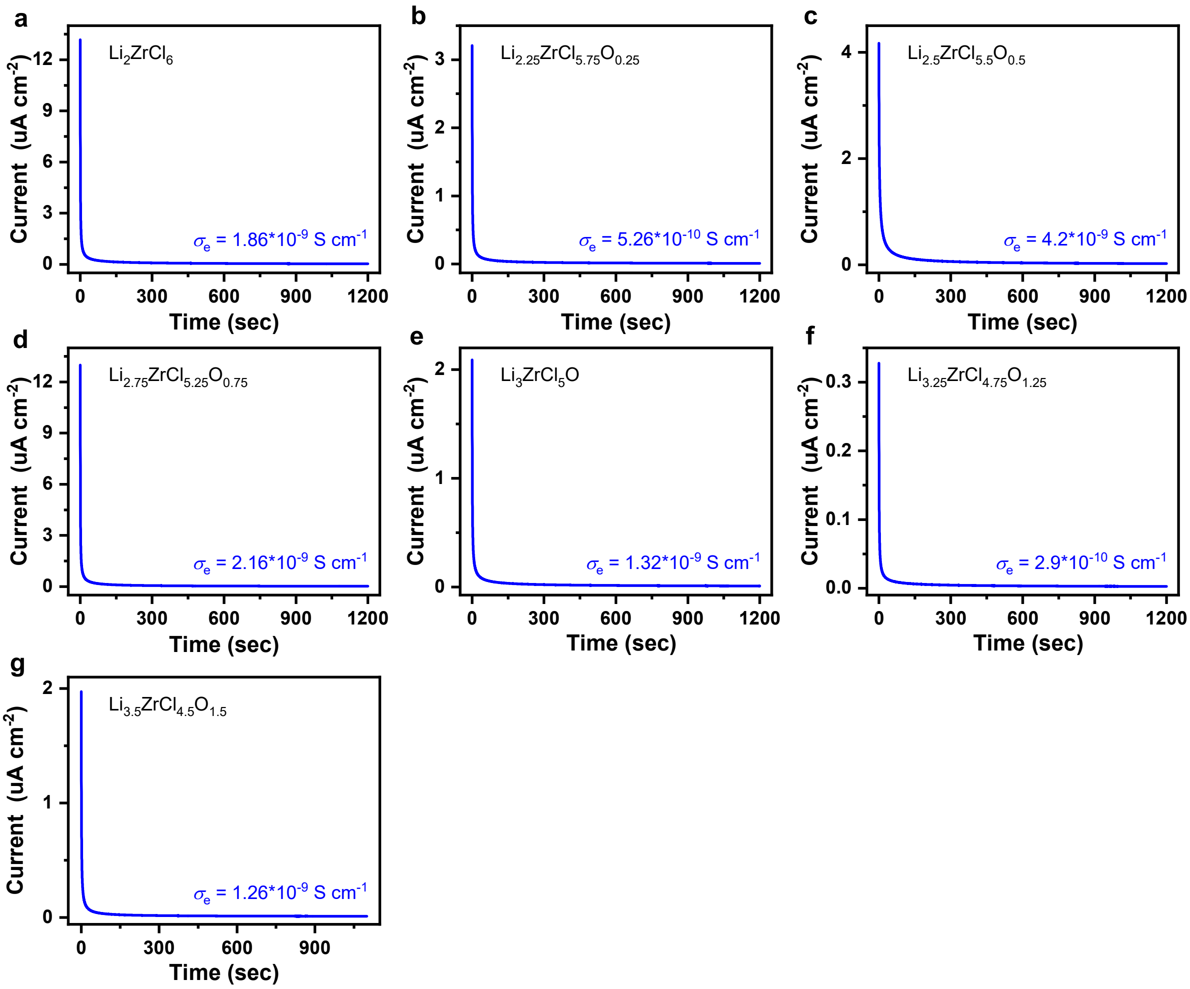

Supplementary Fig. 11. The transient current behavior under DC bias for the $Li_{2+x}ZrCl_{6-x}O_x$ materials with x = 0 (**a**), 0.25 (**b**), 0.50 (**c**), 0.75 (**d**), 1.0 (**e**), 1.25 (**f**), and 1.50 (**g**) with stainless steel electrodes at 25 °C.

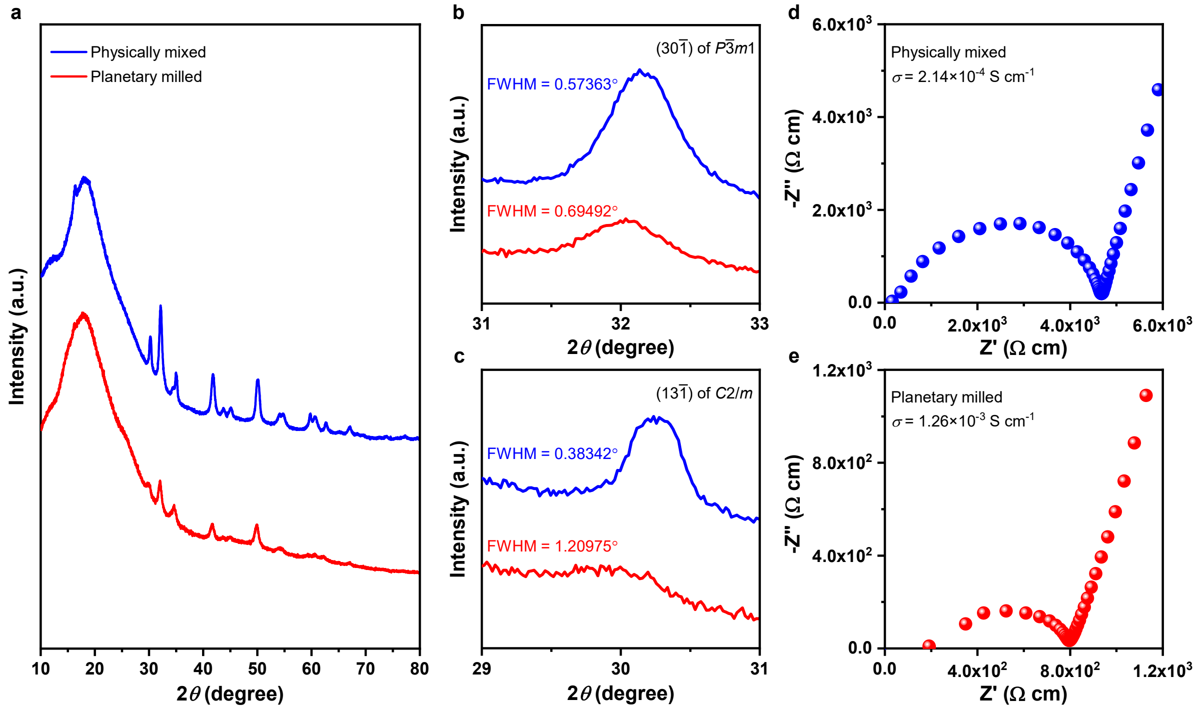

Supplementary Fig. 12. **a**, XRD patterns of the powders prepared by milling the separately synthesized Li_2ZrCl_6 and $Li_4ZrCl_4O_2$ together in two different ways; one is physically mixed through manual grinding for 30 minutes, and the other is planetary milled with the parameters used for the mechaochemical synthesis. For both powders, the Li_2ZrCl_6:$Li_4ZrCl_4O_2$ molar ratio is 75:25, equivalent to an overall composition of $Li_{2.5}ZrCl_{5.5}O_{0.5}$. No smoothening was conducted to the data displayed here. **b**,**c**, The $(30\bar{1})$ peak of the $P\bar{3}m1$ phase (**b**) and the $(13\bar{1})$ peak of the *C2/m* phase (**c**) for the physically mixed and planetary milled powders. FWHM stands for full width of half maximum. **d**,**e**, Nyquist plots of the physically mixed (**d**) and planetary milled powders (**e**) at 25 °C. The EIS measurement was performed using the cold-pressed pellet with 10 mm diameter sandwiched between two stainless steel electrodes, under an external pressure of 2.8 tons.

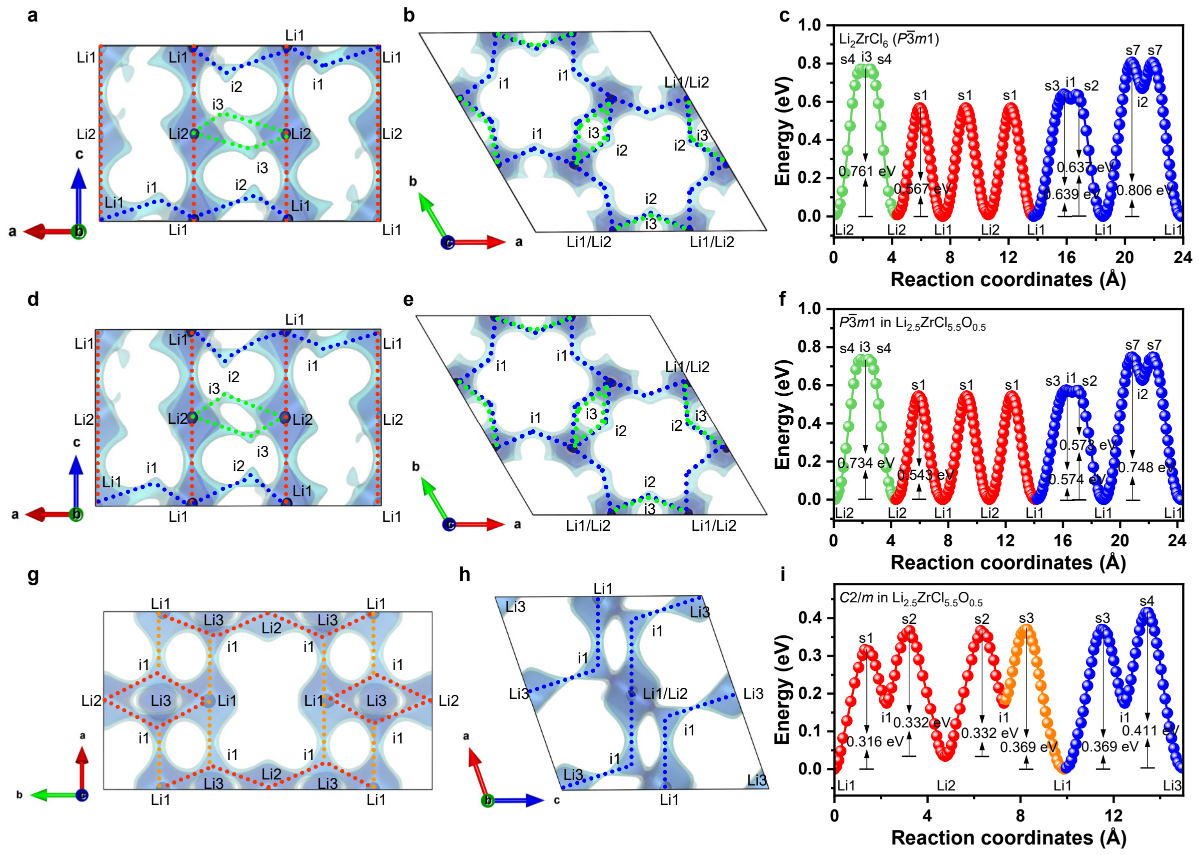

Supplementary Fig. 13. BVSE analysis of Li-ion migration in the crystalline phases of Li_2ZrCl_6 and $Li_{2.5}ZrCl_{5.5}O_{0.5}$. **a**,**b**,**d**,**e**,**g**,**h**, Li-ion migration pathways of Li_2ZrCl_6 with the $P\bar{3}m1$ structure (**a,b**), along with those of the $P\bar{3}m1$ phase (**d,e**) and the *C2/m* phase (**g,h**) in $Li_{2.5}ZrCl_{5.5}O_{0.5}$. **c**,**f**,**i**, Energy profiles of the Li-ion migration pathways in Li_2ZrCl_6 (**c**), along with those for the $P\bar{3}m1$ phase (**f**) and the *C2/m* phase (**i**) in $Li_{2.5}ZrCl_{5.5}O_{0.5}$. Each pathway in **a**–**b**, **d**–**e**, and **g**–**h** corresponds to the energy profile of the same color in **c**, **f**, and **i**, respectively. The data presented here were derived from the Rietveld refinement results of Li_2ZrCl_6 (Supplementary Table 1) and $Li_{2.5}ZrCl_{5.5}O_{0.5}$ (Supplementary Table 3).

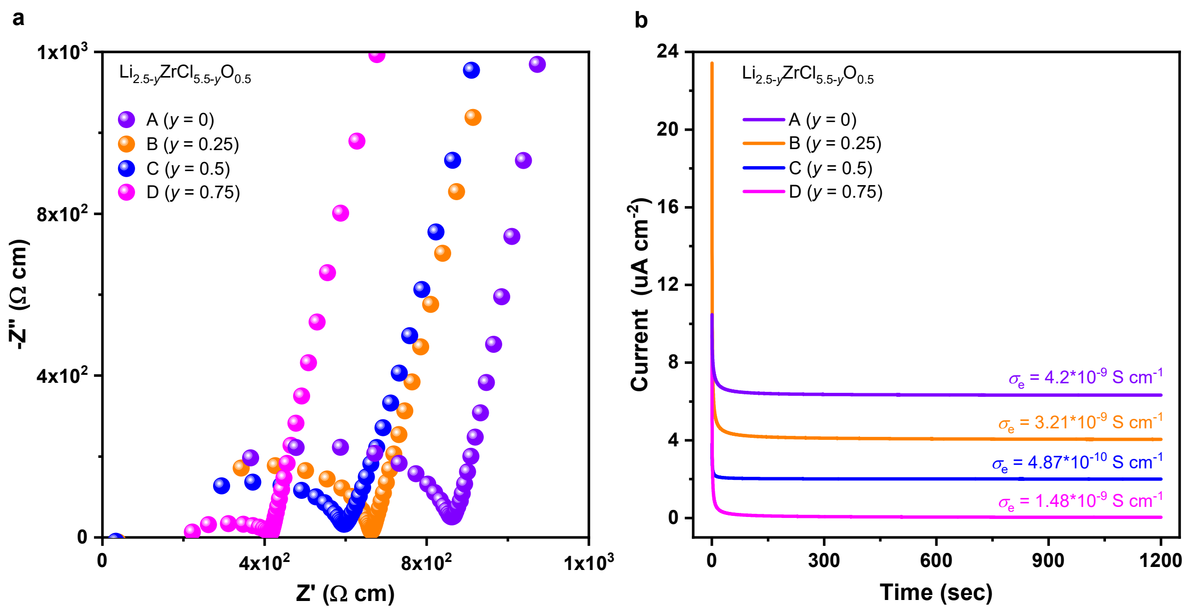

Supplementary Fig. 14. **a**, Nyquist plots of $Li_{2.5\text{-}y}ZrCl_{5.5\text{-}y}O_{0.5}$ with different compositions at 25 °C. The EIS measurement was performed using the cold-pressed pellet with 10 mm diameter sandwiched between two stainless steel electrodes, under an external pressure of 2.8 tons. **b**, The transient current behavior under DC bias for $Li_{2.5\text{-}y}ZrCl_{5.5\text{-}y}O_{0.5}$ with stainless steel electrodes at 25 °C. For clarity, the data for the compositions with y = 0, 0.25, and 0.5 are shifted vertically by 6, 4, and 2 μA cm^{-2}, respectively.

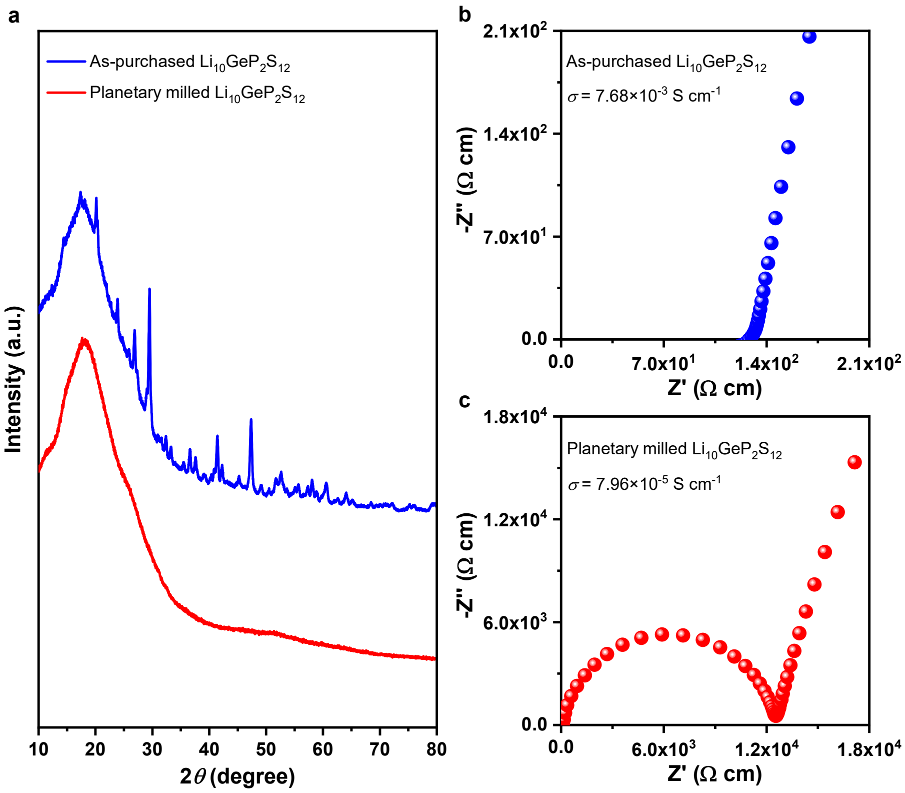

Supplementary Fig. 15. a, XRD patterns of the as-purchased $Li_{10}GeP_2S_{12}$ and the $Li_{10}GeP_2S_{12}$ that were planetary milled after purchase (milling conditions identical with those for synthesizing LZCO). The broad hump below 30° comes from the Kapton film that was used to prevent air exposure. No smoothening was conducted to the data displayed here. **b,c,** Nyquist plots of the as-purchased (**b**) and planetary milled $Li_{10}GeP_2S_{12}$ (**c**) at 25 °C. The EIS measurement was performed using the cold-pressed pellet with 10 mm diameter sandwiched between two stainless steel electrodes, under an external pressure of 2.8 tons.

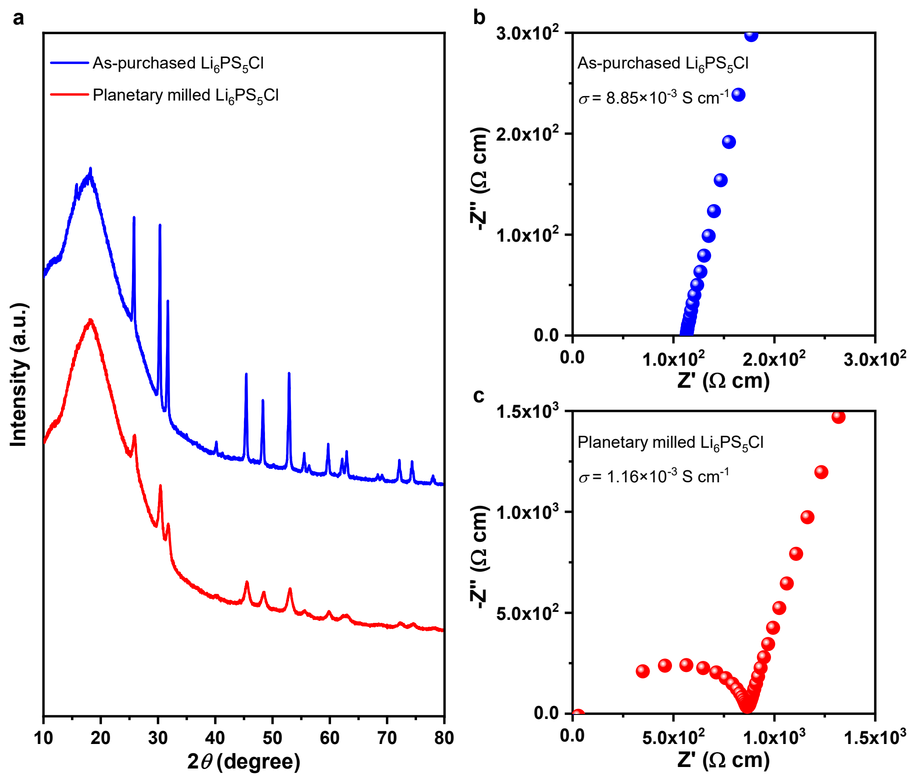

Supplementary Fig. 16. a, XRD patterns of the as-purchased Li_6PS_5Cl and the Li_6PS_5Cl that were planetary milled after purchase (milling conditions identical with those for synthesizing LZCO). The broad hump below 30° comes from the Kapton film that was used to prevent air exposure. No smoothening was conducted to the data displayed here. **b,c,** Nyquist plots of the as-purchased (**b**) and planetary milled Li_6PS_5Cl (**c**) at 25 °C. The EIS measurement was performed using the cold-pressed pellet with 10 mm diameter sandwiched between two stainless steel electrodes, under an external pressure of 2.8 tons.

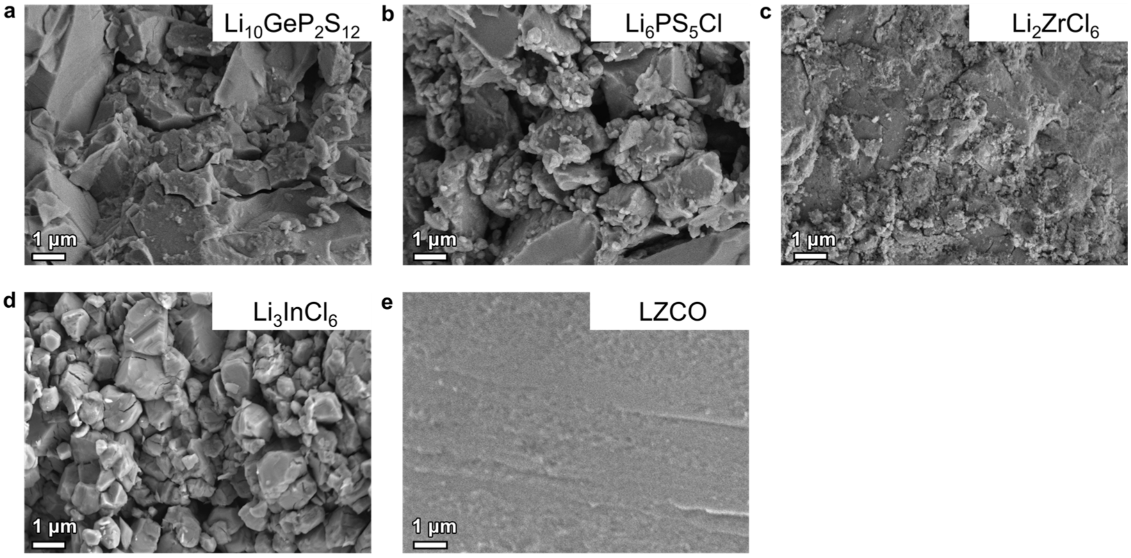

Supplementary Fig. 17. a–e, SEM images taken from the cross sections of the cold-pressed $Li_{10}GeP_2S_{12}$ (**a**), Li_6PS_5Cl (**b**), Li_2ZrCl_6 (**c**), Li_3InCl_6 (**d**), and LZCO pellets (**e**). All the pellets were fabricated under 300 MPa.

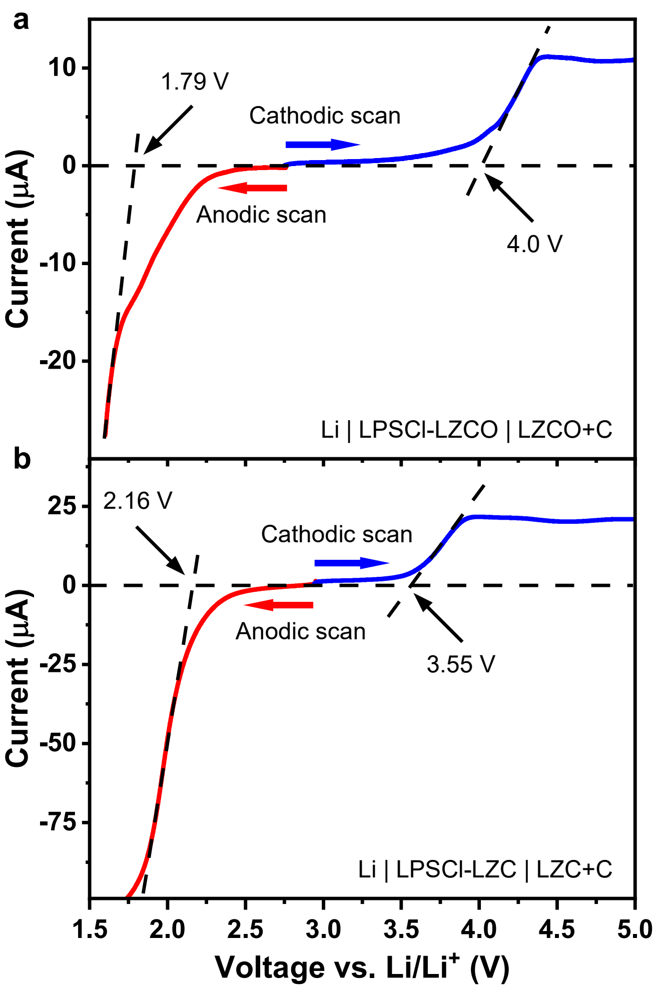

Supplementary Fig. 18. a,**b**, LSV curves of the Li | LPSCl-LZCO | LZCO + C (**a**) and the Li | LPSCl-LZC | LZC + C cells (**b**) at 0.1 mV s^{-1}. The measurements were conducted at 25 °C and 0.1 tons of stacking pressure.

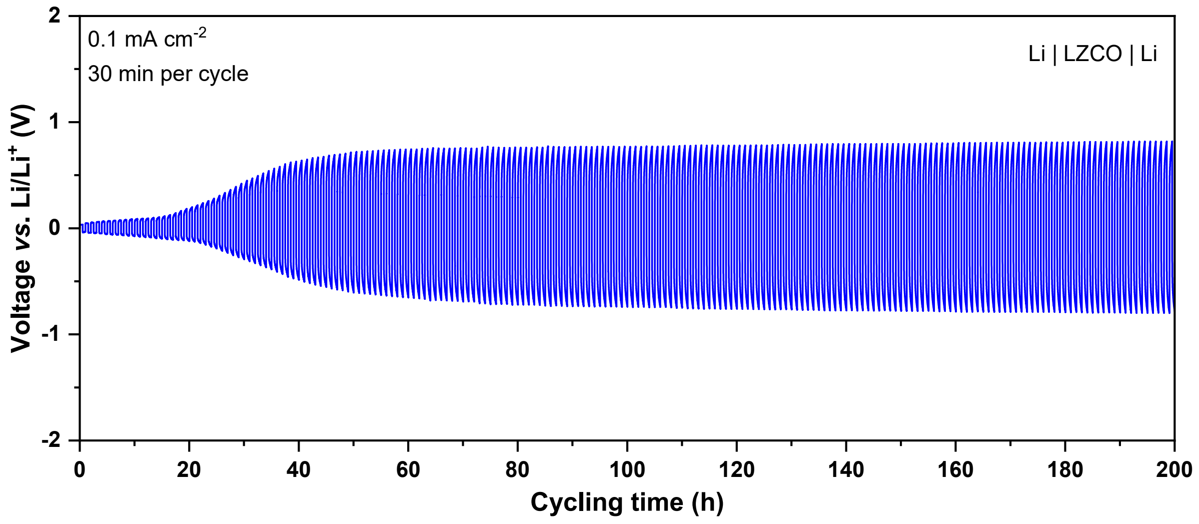

Supplementary Fig. 19. Galvanostatic cycling profiles of the symmetric Li | LZCO | Li cell at 0.1 mA cm^{-2} (30 minutes per cycle). The measurement was conducted at 25 °C and 0.1 tons of stacking pressure.

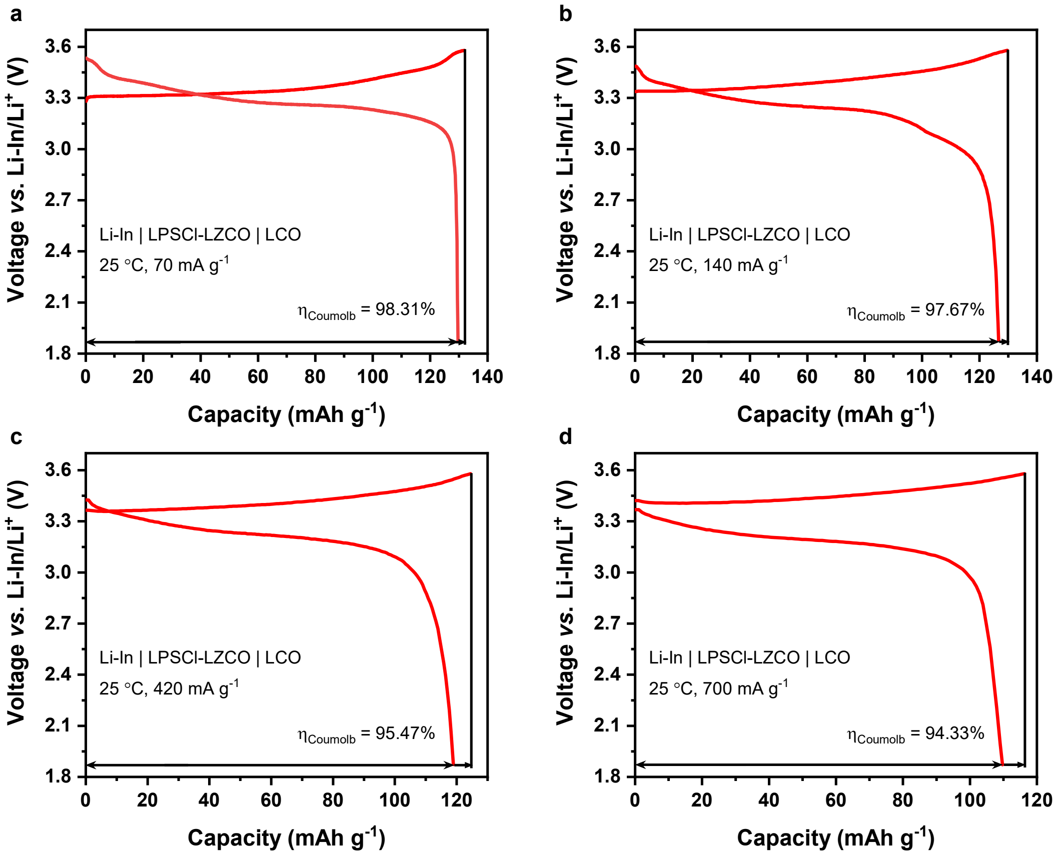

Supplementary Fig. 20. a–d, Initial charge and discharge voltage profiles of the Li-In | LPSCl-LZCO | LCO cell cycled between 1.88 and 3.58 V *vs*. Li-In/Li^{+} under 70 mA g^{-1} (**a**), 140 mA g^{-1} (**b**), 420 mA g^{-1} (**c**), and 700 mA g^{-1} (**d**) at 25 °C and 1.5 tons of stacking pressure, with the Coulombic efficiencies η_{Coulomb} denoted. The data at each rate were collected using a fresh cell.

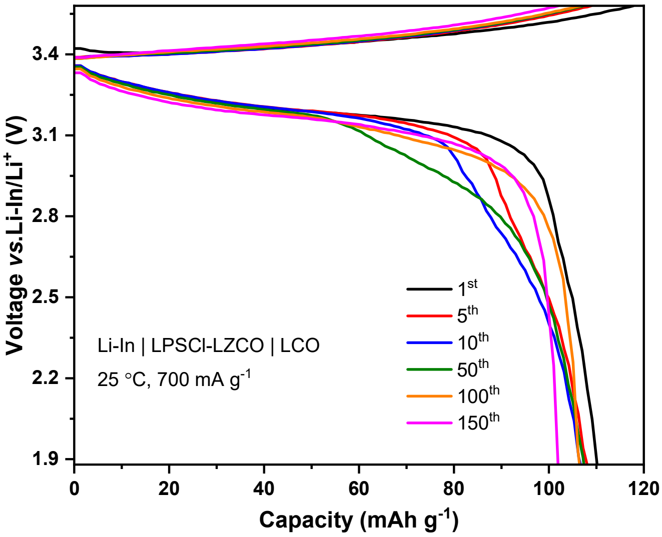

Supplementary Fig. 21. Charge and discharge voltage profiles of the Li-In | LPSCl-LZCO | LCO cell during the long-term cycling between 1.88 and 3.58 V *vs*. Li-In/Li^+ under 700 mA g^{-1} at 25 °C and 1.5 tons of stacking pressure.

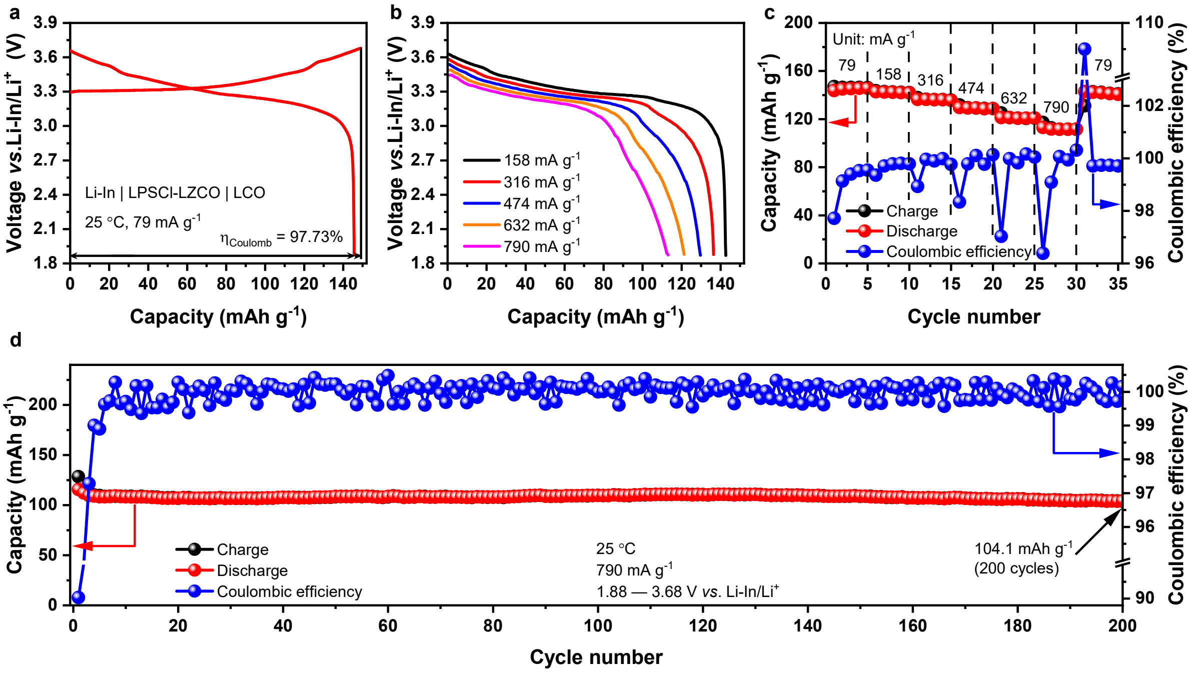

Supplementary Fig. 22. a, Initial charge and discharge voltage profiles of the Li-In | LPSCl-LZCO | LCO cell at 79 mA g^{-1}, with the Coulombic efficiency η_{Coulomb} denoted. **b**,**c**, Rate capability at 79, 158, 316, 474, 632 and 790 mA g^{-1}. **d**, Long-term cycling performance at 790 mA g^{-1}. All the cycling tests were conducted between 1.88 and 3.68 V *vs*. Li-In/Li$^+$ at 25 °C and 1.5 tons of stacking pressure.

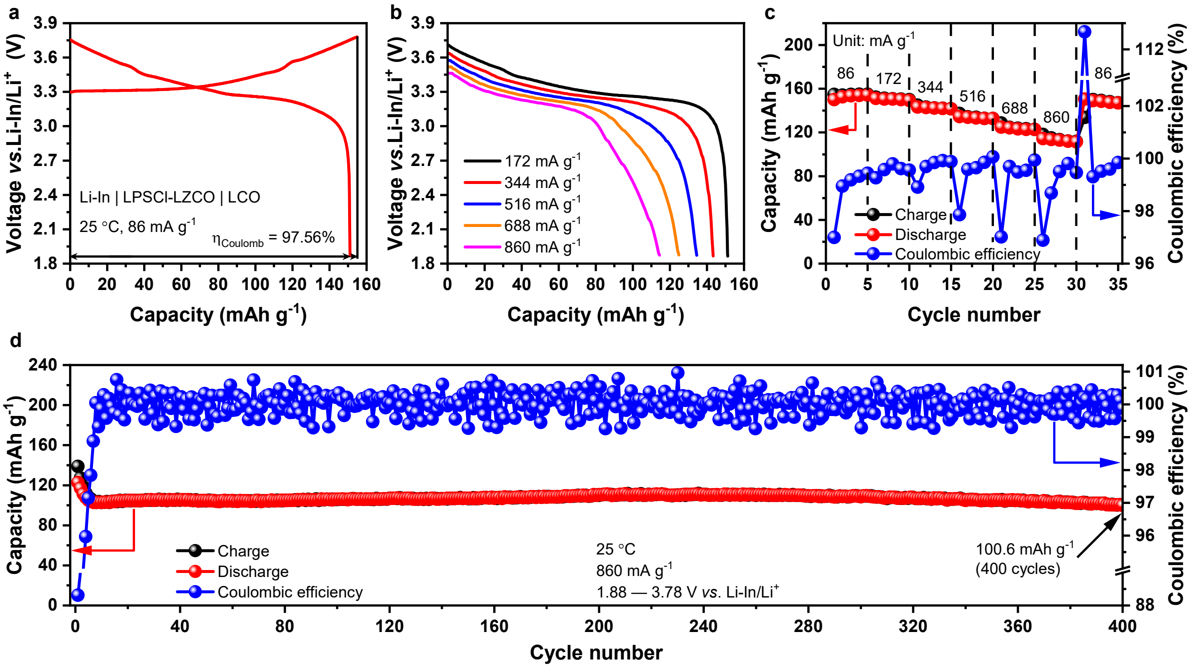

Supplementary Fig. 23. a, Initial charge and discharge voltage profiles of the Li-In | LPSCl-LZCO | LCO cell at 86 mA g^{-1}, with the Coulombic efficiency η_{Coulomb} denoted. **b**,**c**, Rate capability at 86, 172, 344, 516, 688 and 860 mA g^{-1}. **d**, Long-term cycling performance at 860 mA g^{-1}. All the cycling tests were conducted between 1.88 and 3.78 V *vs*. Li-In/Li^{+} at 25 °C and 1.5 tons of stacking pressure.

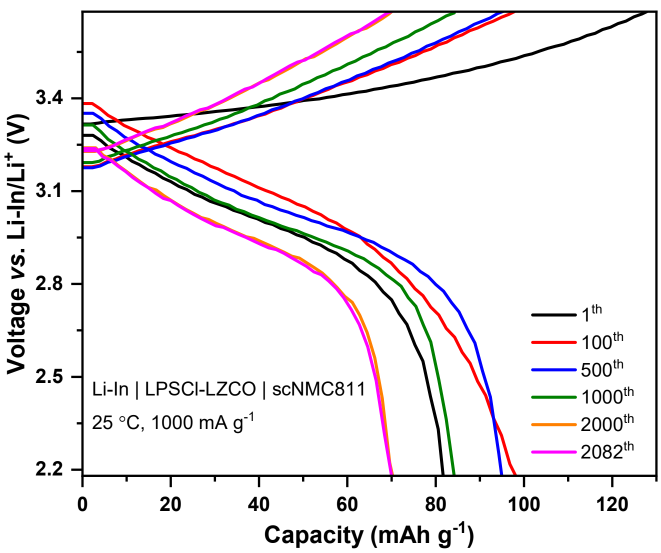

Supplementary Fig. 24. Charge and discharge voltage profiles of the Li-In | LPSCl-LZCO | scNMC811 cell during the long-term cycling between 2.18 and 3.68 V *vs*. Li-In/Li^+ under 1000 mA g^{-1} at 25 °C and 1.5 tons of stacking pressure.

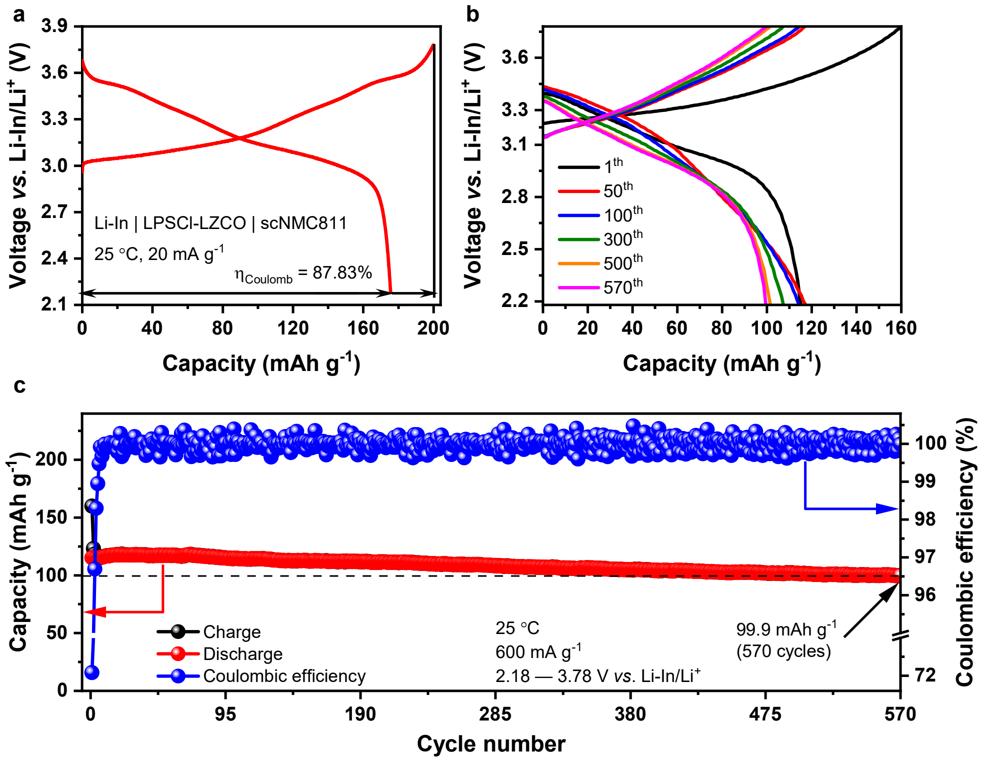

Supplementary Fig. 25. **a**, Initial charge and discharge voltage profiles of the Li-In | LPSCl-LZCO | scNMC811 cell cycled between 2.18 and 3.78 V *vs*. Li-In/Li^+ under 20 mA g^{-1} at 25 °C and 1.5 tons of stacking pressure. The Coulombic efficiency η_{Coulomb} is also denoted. **b**, Charge and discharge voltage profiles of the Li-In | LPSCl-LZCO | scNMC811 cell during the long-term cycling between 2.18 and 3.78 V *vs*. Li-In/Li^+ under 600 mA g^{-1} at 25 °C and 1.5 tons of stacking pressure. **c**, Capacities and Coulombic efficiencies at different cycles for the long-term cycling shown in **b**.

Supplementary Table 1. Rietveld refinement results from the XRD pattern of Li_2ZrCl_6. The space group is $P\overline{3}m1$; the refined lattice parameters are a = 10.970(4) Å and c = 5.9427(13) Å.

Atom	x	y	z	Occupancy	Site	U_{iso} (Å^2)
Li1	0.53(9)	0	0	0.63(6)	6*g*	0.035(11)
Li2	0.361(9)	0	0.5	0.37(40)	6*h*	0.03(5)
Cl1	0.1102(6)	-0.1102(6)	0.7781(17)	1	6*i*	0.0196(30)
Cl2	0.2281(5)	-0.2281(5)	0.2470(15)	1	6*i*	0.041(4)
Cl3	0.4547(5)	-0.4547(5)	0.6936(14)	1	6*i*	0.0143(25)
Zr1	0	0	0	0.597(15)	1*a*	0.010(3)
Zr2	1/3	2/3	0.5108(15)	0.547(9)	2*d*	0.0027(25)
Zr3	0	0	0.5	0.571(13)	1*b*	0.0001(25)
Zr4	1/3	2/3	0.949(4)	0.369(1)	2*d*	0.003(4)

Supplementary Table 2. Rietveld refinement results from the XRD pattern of $Li_{2.25}ZrCl_{5.75}O_{0.25}$. The space group is $P\overline{3}m1$; the refined lattice parameters are *a* = 10.9760(18) Å and *c* = 5.9427(6) Å.

Atom	*x*	*y*	*z*	Occupancy	Site	U_{iso} (Å^2)
Li1	0.282(16)	0	0	0.22(3)	6*g*	0.04(6)
Li2	0.353(30)	0	0.5	0.905(1)	6*h*	0.036(27)
Cl1	0.1094 (30)	-0.1094(30)	0.7605(7)	0.933(17)	6*i*	0.012(4)
Cl2	0.2336(4)	-0.2336(4)	0.2837(16)	0.941(14)	6*i*	0.012(4)
Cl3	0.4521(7)	-0.4521(7)	0.7843(12)	1	6*i*	0.0116(30)
O1	0.1094(30)	-0.1094(30)	0.7605(7)	0.067(17)	6*i*	0.04(5)
O2	0.2336(4)	-0.2336(4)	0.2837(16)	0.059(14)	6*i*	0.01(21)
O3	0.4521(7)	-0.4521(7)	0.7843(12)	0	6*i*	0.01(13)
Zr1	0	0	0	0.626(9)	1*a*	0.015(5)
Zr2	1/3	2/3	0.5491(21)	0.489(5)	2*d*	0.0082(27)
Zr3	0	0	0.5	0.615(8)	1*b*	0.005(4)
Zr4	1/3	2/3	0.9609(25)	0.391(7)	2*d*	0.001(4)

Supplementary Table 3. Rietveld refinement results from the XRD pattern of $Li_{2.5}ZrCl_{5.5}O_{0.5}$. For Phase I, the space group is $P\bar{3}m1$; the refined lattice parameters are a = 10.937(9) Å and c = 6.022(6) Å. For Phase II, the space group is $C2/m$; the refined lattice parameters are a = 6.310(10) Å, b = 11.017(5) Å, c = 6.285(12) Å, and β = 109.314(31)°.

Phase	Atom	*x*	*y*	*z*	Occupancy	Site	U_{iso} (Å^2)
	Li1	0.311(19)	0	0	0.53(12)	6*g*	0.04(12)
	Li2	0.337(25)	0	0.5	0.62(10)	6*h*	0.03(6)
	Cl1	0.1061(16)	-0.1061(16)	0.7514(21)	0.95(4)	6*i*	0.012(5)
	Cl2	0.2283(10)	-0.2283(10)	0.2811(29)	0.923(22)	6*i*	0.012(4)
	Cl3	0.4419(10)	-0.4419(10)	0.7676(26)	0.977(5)	6*i*	0.012(3)
Phase I	O1	0.1061(16)	-0.1061(16)	0.7514(21)	0.05(4)	6*i*	0.03(17)
($P\bar{3}m1$)	O2	0.2283(10)	-0.2283(10)	0.2811(29)	0.077(22)	6*i*	0.11(29)
	O3	0.4419(10)	-0.4419(10)	0.7676(26)	0.023(5)	6*i*	0.0(3)
	Zr1	0	0	0	0.649(24)	1*a*	0.006(10)
	Zr2	1/3	2/3	0.520(4)	0.565(32)	2*d*	0.007(7)
	Zr3	0	0	0.5	0.709(17)	1*b*	0.008(7)
	Zr4	1/3	2/3	0.939(8)	0.256(1)	2*d*	0.005(15)
	Li1	0	0.181(22)	0.5	0.58(24)	4*h*	0.03(9)
	Li2	0.5	0	0.5	0.2(4)	2*d*	0.02(5)
	Li3	0.5	0.822(15)	0	0.77(5)	4*g*	0.01(6)
Phase II	Cl1	0.2388(24)	0.8385(16)	0.2361(29)	0.825(19)	8*j*	0.014(11)
($C2/m$)	Cl2	0.760(3)	0	0.232(3)	0.90(9)	4*i*	0.014(14)
	O1	0.2388(24)	0.8385(16)	0.2361(29)	0.175(19)	8*j*	0.00(11)
	O2	0.760(3)	0	0.232(3)	0.10(9)	4*i*	0.00(29)
	Zr1	0	0	0	1	2*a*	0.012(5)

Supplementary Table 4. Rietveld refinement results from the XRD pattern of $Li_{2.75}ZrCl_{5.25}O_{0.75}$. For Phase I, the space group is $P\bar{3}m1$; the refined lattice parameters are a = 11.072(12) Å and c = 5.822(5) Å. For Phase II, the space group is $C2/m$; the refined lattice parameters are a = 6.269(13) Å, b = 11.032(7) Å, c = 6.306(13) Å, and β = 109.26(4)°.

Phase	Atom	x	y	z	Occupancy	Site	U_{iso} (Å^2)
Phase I ($P\bar{3}m1$)	Li1	0.25(3)	0	0	0.55(13)	6*g*	0.03(9)
	Li2	0.302(31)	0	0.5	0.60(48)	6*h*	0.03(5)
	Cl1	0.1009(18)	-0.1009(18)	0.7510(16)	0.92(6)	6*i*	0.012(5)
	Cl2	0.2308(24)	-0.2308(24)	0.2797(17)	0.96(23)	6*i*	0.012(4)
	Cl3	0.4419(16)	-0.4419(16)	0.7664(21)	0.97(6)	6*i*	0.012(4)
	O1	0.1009(18)	-0.1009(18)	0.7510(16)	0.08(6)	6*i*	0.05(21)
	O2	0.2308(24)	-0.2308(24)	0.2797(17)	0.04(23)	6*i*	0.0(5)
	O3	0.4419(16)	-0.4419(16)	0.7664(21)	0.03(6)	6*i*	0.0(3)
	Zr1	0	0	0	0.65(3)	1*a*	0.009(12)
	Zr2	1/3	2/3	0.531(3)	0.57(3)	2*d*	0.007(8)
	Zr3	0	0	0.5	0.71(19)	1*b*	0.007(8)
	Zr4	1/3	2/3	0.934(9)	0.25(1)	2*d*	0.007(20)
Phase II ($C2/m$)	Li1	0	0.12(5)	0.5	0.31(29)	4*h*	0.03(12)
	Li2	0.5	0	0.5	0.28(3)	2*d*	0.02(12)
	Li3	0.5	0.881(18)	0	1	4*g*	0.01(4)
	Cl1	0.242(3)	0.8446(19)	0.238(4)	0.83(3)	8*j*	0.012(8)
	Cl2	0.760(5)	0	0.244(4)	0.89(1)	4*i*	0.014(9)
	O1	0.242(3)	0.8446(19)	0.238(4)	0.17(3)	8*j*	0.03(8)
	O2	0.760(5)	0	0.244(4)	0.11(1)	4*i*	0.04(31)
	Zr1	0	0	0	1	2*a*	0.012(5)

Supplementary Table 5. Rietveld refinement results from the XRD pattern of Li_3ZrCl_5O. The space group is *C*2/*m*; the refined lattice parameters are a = 6.295(5) Å, b = 10.9287(16) Å, c = 6.329(5) Å, and β = 110.013(14)°.

Atom	*x*	*y*	*z*	Occupancy	Site	U_{iso} (Å^2)
Li1	0	0.169(7)	0.5	0.37(5)	4*h*	0.02(4)
Li2	0.5	0	0.5	0.26(6)	2*d*	0.01(12)
Li3	0.5	0.8388(26)	0	1	4*g*	0.010(16)
Cl1	0.2408(4)	0.8414(4)	0.2322(7)	0.833(5)	8*j*	0.0124(18)
Cl2	0.7547(7)	0	0.2276(8)	0.834(0)	4*i*	0.0126(23)
O1	0.2408(4)	0.8414(4)	0.2322(7)	0.167(5)	8*j*	0.011(26)
O2	0.7547(7)	0	0.2276(8)	0.166(0)	4*i*	0.01(4)
Zr1	0	0	0	1	2*a*	0.0127(12)

Supplementary Table 6. Rietveld refinement results from the XRD pattern of $Li_{3.25}ZrCl_{4.75}O_{1.25}$. The space group is *C*2/*m*; the refined lattice parameters are a = 6.2066(28) Å, b = 10.9063(11) Å, c = 6.246(3) Å, and β = 109.131(8)°.

Atom	*x*	*y*	*z*	Occupancy	Site	U_{iso} (Å^2)
Li1	0	0.1610(29)	0.5	0.55(3)	4*h*	0.014(24)
Li2	0.5	0	0.5	0.15(8)	2*d*	0.02(7)
Li3	0.5	0.8223(17)	0	1	4*g*	0.012(11)
Cl1	0.2396(4)	0.8380(30)	0.2362(5)	0.792(4)	8*j*	0.0129(17)
Cl2	0.7565(7)	0	0.2323(5)	0.791(3)	4*i*	0.0122(17)
O1	0.2396(4)	0.8380(30)	0.2362(5)	0.208(4)	8*j*	0.013(19)
O2	0.7565(7)	0	0.2323(5)	0.209(3)	4*i*	0.010(20)
Zr1	0	0	0	1	2*a*	0.0127(12)

Supplementary Table 7. Rietveld refinement results from the XRD pattern of $Li_{3.5}ZrCl_{4.5}O_{1.5}$. The space group is *C*2/*m*; the refined lattice parameters are *a* = 6.257(5) Å, *b* = 10.9334(17) Å, *c* = 6.283(4) Å, and *β* = 109.111(13)°.

Atom	*x*	*y*	*z*	Occupancy	Site	U_{iso} (Å^2)
Li1	0	0.175(5)	0.5	0.57(6)	4*h*	0.01(7)
Li2	0.5	0	0.5	0.36(8)	2*d*	0.01(17)
Li3	0.5	0.8158(26)	0	1	4*g*	0.01(3)
Cl1	0.2340(8)	0.8412(4)	0.2424(8)	0.755(24)	8*j*	0.012(5)
Cl2	0.7616(11)	0	0.2326(14)	0.740(3)	4*i*	0.013(5)
O1	0.2340(8)	0.8412(4)	0.2424(8)	0.245(24)	8*j*	0.001(26)
O2	0.7616(11)	0	0.2326(14)	0.260(3)	4*i*	0.00(7)
Zr1	0	0	0	1	2*a*	0.013(5)

Supplementary Table 8. Rietveld refinement results from the XRD pattern of $Li_4ZrCl_4O_2$. The space group is *C*2/*m*; the refined lattice parameters are *a* = 6.2929(22) Å, *b* = 10.9030(10) Å, *c* = 6.2871(24) Å, and *β* = 109.515(5)°.

Atom	*x*	*y*	*z*	Occupancy	Site	U_{iso} (Å^2)
Li1	0	0.1715(32)	0.5	0.824(10)	4*h*	0.012(8)
Li2	0.5	0	0.5	0.35(30)	2*d*	0.02(3)
Li3	0.5	0.8264(20)	0	1	4*g*	0.015(12)
Cl1	0.2395(7)	0.8391(28)	0.2363(8)	0.666(11)	8*j*	0.013(4)
Cl2	0.7591(11)	0	0.2321(10)	0.669(5)	4*i*	0.012(6)
O1	0.2395(7)	0.8391(28)	0.2363(8)	0.334(11)	8*j*	0.012(27)
O2	0.7591(11)	0	0.2321(10)	0.331(5)	4*i*	0.01(4)
Zr1	0	0	0	1	2*a*	0.0127(7)

Supplementary Note 1. Cost analysis

The raw materials cost of the solid electrolyte was estimated based upon the prices of the chemicals needed for synthesis in 1000-kg purchase. Such chemical prices were inferred from the recent laboratory-scale prices[1] using the method proposed by Hart et al.[2]. Specifically, Hart et al. found that the unit price, *P*, and the purchase quantity, *Q*, of the chemicals satisfy this general relationship:

$$\log_{10} P = \log_{10} a + b \times \log_{10} Q$$

where a and b are constants for a given chemical. In this way, the unit price for the purchase in 1000-kg quantity can be inferred through linear extrapolation based on a series of unit prices *P* and quantities *Q* for the laboratory-scale purchase. The *P* and *Q* values of the chemicals used for the cost analysis here were adopted from a recent publication[1].

Supplementary References

1. Wang, K. *et al.* A cost-effective and humidity-tolerant chloride solid electrolyte for lithium batteries. *Nat. Commun.* **12,** 4410 (2021).

2. Hart, P. & Sommerfeld, J. Cost estimation of specialty chemicals from laboratory-scale prices. *Cost Eng.* **39,** 31-35 (1997).
